# CD30 influences germinal center B-cell dynamics and the expansion of IgG1-switched B cells

**DOI:** 10.1038/s41423-024-01219-w

**Published:** 2024-10-17

**Authors:** Yan Wang, Ursula Rambold, Petra Fiedler, Tea Babushku, Claas L. Tapken, Kai P. Hoefig, Thomas P. Hofer, Heiko Adler, Ali Önder Yildirim, Lothar J. Strobl, Ursula Zimber-Strobl

**Affiliations:** 1 Research Unit Gene Vectors, Research Group B-Cell Development and Activation, Helmholtz Center Munich, German Research Center for Environmental Health, Munich, Germany; 2 Institute of Asthma and Allergy Prevention, Helmholtz Center Munich, German Research Center for Environmental Health, Neuherberg, Germany; 3 Member of the German Center of Lung Research (DZL), Munich, Germany; 4 Institute of Lung Health and Immunity (LHI), Helmholtz Center Munich, Comprehensive Pneumology Center (CPC-M), Neuherberg, Germany; 5 Research Unit Molecular Immune Regulation, Helmholtz Center Munich, Munich, Germany; 6 Immunoanalytics - Research Group Tissue Control of Immunocytes, Helmholtz Center Munich, Munich, Germany; 7 Walther Straub Institute of Pharmacology and Toxicology, Ludwig-Maximilians-University Munich, Munich, Germany

**Keywords:** B lymphocytes, Germinal center reaction, CD30, Conditional mice, CD30L, Senescence associated T cells (SAT cells), Germinal centres, B cells

## Abstract

Initially, identified as a Hodgkin lymphoma marker, CD30 was subsequently detected on a subset of human B cells within and around germinal centers (GCs). While CD30 expression is typically restricted to a few B cells, expansion of CD30-expressing B cells occurs in certain immune disorders and during viral infections. The role of CD30 in B cells remains largely unclear. To address this gap in knowledge, we established a conditional CD30-knockin mouse strain. In these mice, B-cell-specific CD30 expression led to a normal B-cell phenotype in young mice, but most aged mice exhibited significant expansion of B cells, T cells and myeloid cells and increased percentages of GC B cells and IgG1-switched cells. This may be driven by the expansion of CD4^+^ senescence-associated T cells and T follicular helper cells, which partially express CD30-L (CD153) and may stimulate CD30-expressing B cells. Inducing CD30 expression in antigen-activated B cells accelerates the GC reaction and augments plasma cell differentiation, possibly through the posttranscriptional upregulation of CXCR4. Furthermore, CD30 expression in GC B cells promoted the expansion of IgG1-switched cells, which displayed either a GC or memory-like B-cell phenotype, with abnormally high IgG1 levels compared with those in controls. These findings shed light on the role of CD30 signaling in GC B cells and suggest that elevated CD30^+^ B-cell numbers lead to pathological lymphocyte activation and proliferation.

## Introduction

CD30, also called TNFRSF8, is a member of the tumor necrosis factor receptor (TNF-R) superfamily [1]. CD30 was originally defined as a marker of Hodgkin lymphomas [2, 3], but its expression was later also observed on the surface of other lymphomas, such as diffuse large B-cell lymphomas and primary effusion lymphomas [4–6], and on a subfraction of activated B and T cells. Under physiological conditions, the number of CD30-expressing B cells is very low. CD30^+^ B cells have either a germinal center (GC) or non-GC phenotype and are localized within or at the edge of GCs [7–10].

CD30 is activated by stimulation through a CD30 ligand (CD30-L, CD153), which is membrane-bound and expressed by activated T cells, among other cells [11]. CD30 and CD30-L interact as trimeric structures, resulting in the recruitment of TNF-R-associated factors (TRAF) and the activation of signaling pathways such as the NF-κB, MAPK and JAK/STAT signaling pathways [12–14].

The exact function of CD30 signaling in B cells remains elusive. Inactivation of CD30 in transgenic mice did not yield a clear B-cell phenotype [15]. Subsequent studies demonstrated that CD30 signaling plays a role in the maintenance of GCs and recall of immune responses. However, a pronounced phenotype was observed only in mice with simultaneous inactivation of CD30 and OX-40 [16]. A recent study utilizing a newly established CD30-KO strain confirmed the potential contribution of CD30 signaling during T-cell-dependent (TD)-immune responses [17]. However, in both studies, it remained uncertain whether the immune defect was caused by the inactivation of CD30 in B cells or in T cells.

Recently, the Küppers group performed expression profiling of human CD30^+^ GC and non-GC B cells [10], indicating that the two populations have similar expression profiles, which are distinct from those of conventional GC and post-GC B cells. Both CD30^+^ GC and non-GC B cells share the feature of a strong MYC signature. CD30^+^ B cells with a non-GC B-cell phenotype appear in similar proportions with and without somatic hypermutation, suggesting that they originate from both pre- and post-GC B cells [18]. The authors suggested that these extrafollicular blasts represent either activated memory B cells or recent emigrants from the GC transitioning to plasma cells (PCs). However, the study did not conclusively determine whether CD30 signaling actively controls the differentiation of GC and non-GC B cells or merely serves as an activation marker of B cells. The infrequent occurrence of CD30^+^ cells in the GC and non-GC B-cell fractions (only ~1% of B cells are CD30^+^ in reactive lymph nodes) poses challenges in studying the function of CD30 signaling in these populations. To address this limitation, we generated transgenic mice in which either constitutively active CD30 signaling or ligand-dependent CD30 can be induced in all B cells or specifically in antigen-activated B cells, depending on Cre expression.

Recently, we described the phenotype of mice expressing a constitutively active CD30 receptor in B cells. This was achieved by the expression of a fusion protein consisting of the transmembrane part of the Epstein‒Barr viral latent membrane protein 1 (LMP1) and the cytoplasmic part of CD30 containing the signaling domain (LMP1/CD30). We found that LMP1/CD30 expression inhibits GC formation and instead leads to the expansion of an aberrantly activated B-cell population containing B1b-like cells, plasmablasts and plasma cells [14]. This aberrantly activated B-cell population expanded over time and ultimately resulted in B-cell lymphoma development.

In the present study, we investigated the phenotype of mice expressing a ligand-dependent CD30 receptor (CD30-KI) in B cells. In contrast to LMP1/CD30 mice, B cells from CD30-KI mice depend on interactions with ligand-expressing cells for the induction of CD30 signaling. We demonstrated that the B-cell phenotype of young mice with CD30-expressing B cells is normal. However, as these mice age, there is a significant expansion of B cells, along with an increase in T cells and myeloid cells. This expansion may be driven by reciprocal stimulation between CD30-L-expressing senescence-associated T cells (SA-T cells) and follicular T helper (Tfh) cells, which expand during aging, particularly in mice with CD30-expressing B cells. Moreover, we show that specific activation of CD30 expression in antigen-activated B cells accelerates the entry of pre-GC B cells into the GC, likely due to the more robust and rapid upregulation of CXCR4 surface expression. Additionally, CD30 signaling results in the expansion of IgG1-switched B cells that have either a GC or non-GC B-cell phenotype. These findings demonstrate for the first time a direct contribution of B-cell-specific CD30 expression to the expansion of B cells and T cells as well as to the differentiation of pre-GC and GC B cells. We believe our discoveries will enhance the understanding of diseases characterized by an increased number of CD30-expressing B cells.

## Results

### Generation of a conditional mouse strain expressing CD30 in B cells

**Fig. 1:**
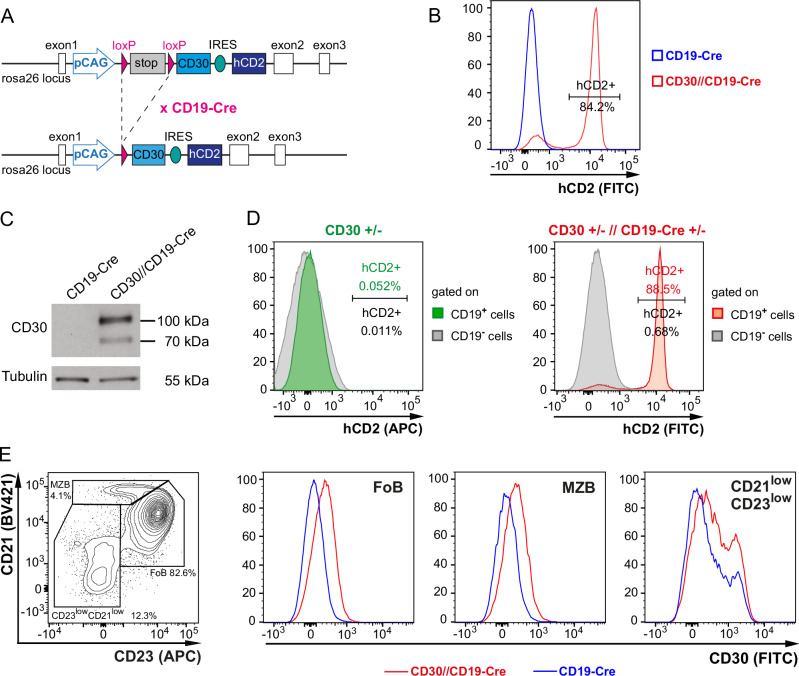
Generation of a mouse strain expressing CD30 on the cell surface of B cells. **A** Schematic representation of the generation of CD30//CD19-Cre mice. cDNA for CD30 was introduced into the *Rosa26* locus via homologous recombination in embryonic stem (ES) cells. Upstream of the CD30 transgene, a loxP-flanked transcriptional/translational stop cassette was inserted. Downstream of the CD30 transgene, an IRES element along with a truncated form of the human hCD2 gene was inserted to enable tracing of the cells in which the stop cassette was deleted. Following removal of the stop cassette, the transgenes are transcriptionally controlled by the CAG promoter. Transgenic ES cells were utilized to generate CD30^stopfl/+^ mice. The expression of CD30 in all B cells was achieved by mating CD30^stopfl^ mice with CD19-Cre mice, resulting in CD30^stopfl/+^//CD19-Cre^+/‒^ (CD30//CD19-Cre) mice. **B** Histogram overlay of hCD2 expression in CD30//CD19-Cre mice (red) compared with that in CD19-Cre control mice (blue), demonstrating that ~85% of all splenic B cells have the stop cassette deleted. A representative of 18 flow cytometric analyses is shown. **C** Protein extracts of splenic B cells were separated via SDS‒PAGE, subjected to Western blotting and detected with an anti-CD30 antibody. The transgenic CD30 protein was detected at the expected size of 110 kDa. The smaller band at ~70 kDa is likely the unglycosylated form of CD30. Tubulin was used as a loading control. **D** Histogram overlays of hCD2 expression in CD19^+^ (green or red, respectively) and CD19^−^ (gray) splenic cells in CD30^stopfl+/−^ (CD30^+/−^) mice and CD30//CD19-Cre mice. Since the hCD2 protein is translated from the same RNA as CD30 it is very likely, that the transgenic CD30 is not expressed in the absence of Cre. **E** To detect transgenic CD30 expression on the surface of different B-cell populations, splenic B cells were separated into three subpopulations. FoB cells (CD23^+^CD21^+^), MZB cells (CD23^−^CD21^high^) and CD23^−^CD21^−^ cells (transitional B cells, B1 cells, and plasmablasts) are gated as illustrated in the FACS plot on the left side. The pregating of the cells is shown in Supplementary Fig. 3a. The histogram overlays illustrate CD30 expression in the indicated populations from CD30//CD19-Cre (red) and CD19-Cre control mice (blue). A representative of 16 flow cytometric analyses is shown

To investigate the role of CD30 in B-cell function, we generated a mouse strain in which CD30 expression could be conditionally induced in different cell types in a manner dependent on the presence of Cre recombinase. This was achieved by inserting a loxP-flanked stop cassette coupled to murine CD30 cDNA into the *Rosa26* locus under the control of the CAG promoter. To be able to detect cells in which the stop cassette was deleted, an IRES hCD2 cassette was inserted downstream of CD30 (CD30^stopfl/+^ mice). To induce CD30 expression in B cells specifically, CD30^stopfl/+^ mice were crossed with CD19-Cre mice to generate CD30^stopfl/+^//CD19-Cre^+/−^ mice (CD30//CD19-Cre mice hereafter) (Fig. 1A). In these mice, most of the splenic B cells had the stop cassette deleted, as shown by their hCD2 (reporter) expression (Fig. 1B). Using Western blotting, the CD30 protein was detected as a 110/70 kDa protein [19] in splenic B cells from CD30//CD19-Cre mice but not in those from CD19-Cre control mice (Fig. 1C). hCD2 was not expressed in B cells from CD30^stopfl/+^ mice (without CD19-Cre) or in non-B cells from CD30//CD19-Cre mice, demonstrating the tightness of the stop cassette (Fig. 1D). FACS analysis revealed an increase in CD30 expression in all the splenic B-cell populations of the CD30//CD19-Cre mice compared with those of the controls (Fig. 1E). The CD30^high^ cells in the CD23^low^CD21^low^ fraction detected in both mutant and control mice could represent preplasmablasts, which physiologically upregulate CD30 [14].

### B-cell-specific CD30 expression results in the expansion of lymphocytes and myeloid cells in aged mice

**Fig. 2:**
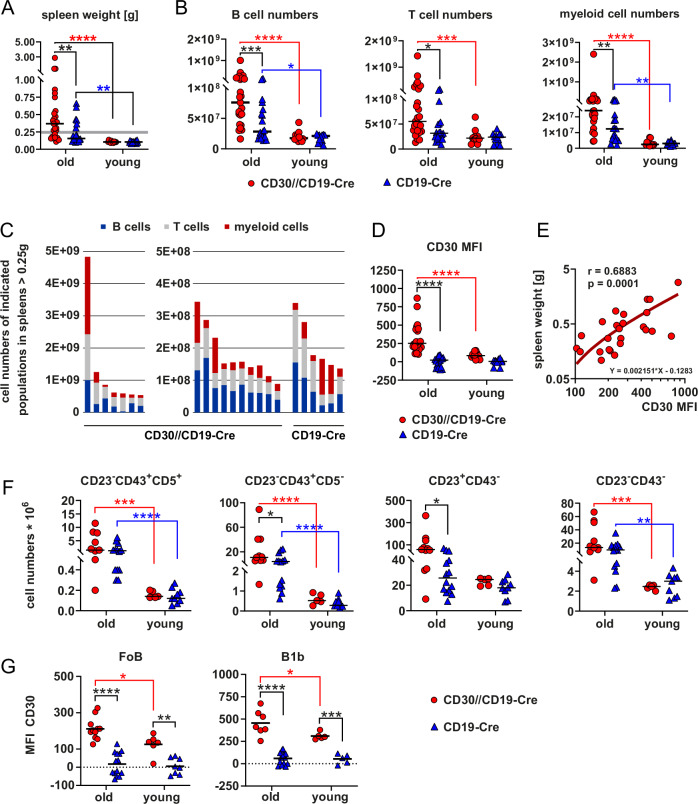
B and T lymphocytes as well as myeloid cells are expanded in the spleens of aged CD30//CD19-Cre mice. **A** Splenic weights (*N* = 17–25 per group) and **B** B-cell numbers, T-cell numbers and numbers of myeloid cells (*N* = 16–25 per group) in young (10–14-week-old) and aged (≥16 months) CD30//CD19-Cre (red) and control mice (blue) are shown. **C** Numbers of B cells, T cells and myeloid cells in the spleens of mice of the indicated genotypes, which have splenomegaly with a weight ≥0.25 g (see also the bold gray line in (**A**)), are indicated in the stacked graphs. The gating strategy for lymphocytes (B and T cells) and myeloid cells is depicted in Supplementary Fig. 1a. The percentages of the different cell populations in the spleen are depicted in a heatmap in Supplementary Fig. 1b. **D** Median fluorescence intensity (MFI) of CD30 on the surface of splenic B cells of the indicated genotypes from young and aged mice (*N* = 11–25 per group). **E** Correlation plot between the splenic weight and the MFI of CD30 on the cell surface of splenic B cells from aged CD30//CD19-Cre mice (*N* = 25). **F** Analysis of the expansion of different B-cell populations in the spleens of aged mice. B cells are categorized into different populations on the basis of their CD43, CD23 and CD5 expression. The gating strategy is illustrated in Supplementary Fig. 3a. The graphs summarize the cell numbers of the gated B-cell populations in mice of the indicated genotypes and ages (*N* = 5–14 per group). The percentages of the different B-cell populations are depicted in Supplementary Fig. 3b. **G** CD30 MFIs in B1b and FoB cells (as gated in Supplementary Fig. 3a) (*N* = 6–14 mice per group). **A**, **B**, **D**, **E** Data from 18 flow cytometric analyses. **F** Data from 9 flow cytometric analyses. **A** + **B** 2-way ANOVA with Sidak’s multiple comparisons test. **D** 2-way ANOVA with Tukey’s multiple comparisons test. **F** 2-way ANOVA with Sidak’s multiple comparisons test. **G** 2-way ANOVA with Tukey’s multiple comparisons test. *P ≤ 0.05, **P ≤ 0.01, ***P ≤ 0.001, ****P ≤ 0.0001

10‒16-week-old mutant and control mice presented comparable splenic weights and B-cell numbers. However, in aged (between 17 and 19 months) CD30//CD19-Cre mice, 68% (17/25 mice) had a splenic weight of more than 0.25 g, whereas this was only the case in 31.6% (6/19 mice) of the control group (Fig. 2A). Both the proportion of mice with an enlarged spleen and the extent of splenic growth were much greater in the CD30//CD19-Cre mice than in the control mice. In both CD19-Cre and CD30//CD19-Cre mice, splenomegaly was attributed not only to an increase in B-cell numbers but also to the expansion of T cells and myeloid cells, which was significantly greater for all populations in old CD30//CD19-Cre mice than in control mice (Fig. 2B). The ratio of expansion among T cells, B cells and myeloid cells varied in different aged CD30//CD19-Cre and control mice with splenomegaly ≥0.25 g (Fig. 2C; Supplementary Fig. 1). In aged CD30//CD19-Cre mice with splenomegaly (17–19 months), the percentages of CD11b^+^ cells (primarily myeloid cells) and CD11c^+^ cells (predominantly dendritic cells) were significantly greater than those in aged control mice and middle-aged (~14 months) CD30//CD19-Cre mice (Supplementary Fig. 2). Among the CD30//CD19-Cre mice, some showed a predominant expansion of CD11b^+^Gr1^+^ granulocytes, whereas others displayed an increase in CD11b^+^Gr1^low^ cells (monocytes and macrophages). Additionally, higher percentages of CD11b^low^Gr1^‒^ cells, likely representing B1 cells and NK cells, were observed in some mice. Overall, these data indicate that mice with splenomegaly exhibit significant variability in the expansion of different hematopoietic cell populations. Aging resulted in significant upregulation of CD30 expression on B cells from CD30//CD19-Cre mice (Fig. 2D), and the extent of CD30 upregulation correlated with the splenic weight of these mice (Fig. 2E). Analysis of the splenic B-cell populations revealed that B1a, B1b and MZB cells reached higher percentages at the expense of FoB cells in both mutant and control aged mice than in young mice (Supplementary Fig. 3b). While CD43^–^CD23^–^ cells (MZB cells and T1 cells) and B1a cells (CD43^+^CD23^–^CD5^+^) presented similar cell numbers in both mouse genotypes of the same age, transgenic CD30 expression led to further increases in the numbers of CD43^+^CD23^–^CD5^–^ (B1b and plasmablasts) and CD43^–^CD23^+^ (FoB) cells compared with those in the controls (Fig. 2F). These findings demonstrate that B1b and/or plasmablasts and FoB cells expand over time. In both FoB and B1b cells, CD30 surface expression levels were significantly greater in CD30//CD19-Cre mice than in control mice. During aging, CD30 levels further increased in both cell populations in the CD30//CD19-Cre mice but remained stable in the controls (Fig. 2G). To study whether CD30-expressing B cells proliferate more than control B cells do, we assessed the percentage of Ki-67^+^ B cells. B1 cells had more Ki-67^+^ cells than FoB cells did, but there was no difference between CD30//CD19-Cre mice and controls within the subpopulations, suggesting comparable proliferation rates in both genotypes (Supplementary Fig. 4a). To confirm these data, we cultured B cells with and without constitutively active CD30 signaling [14], both with and without CD40 stimulation, in vitro (Supplementary Fig. 4b, c). After ex vivo isolation, splenic B cells with active CD30 signaling did not show enhanced proliferation, regardless of CD40 stimulation. However, in the absence of CD40 stimulation, these B cells demonstrated a significant survival advantage. These results suggest that CD30 signaling primarily enhances B-cell survival rather than proliferation.

### Aged CD30//CD19-Cre mice have more spontaneous GCs and more isotype-switched memory B cells

**Fig. 3:**
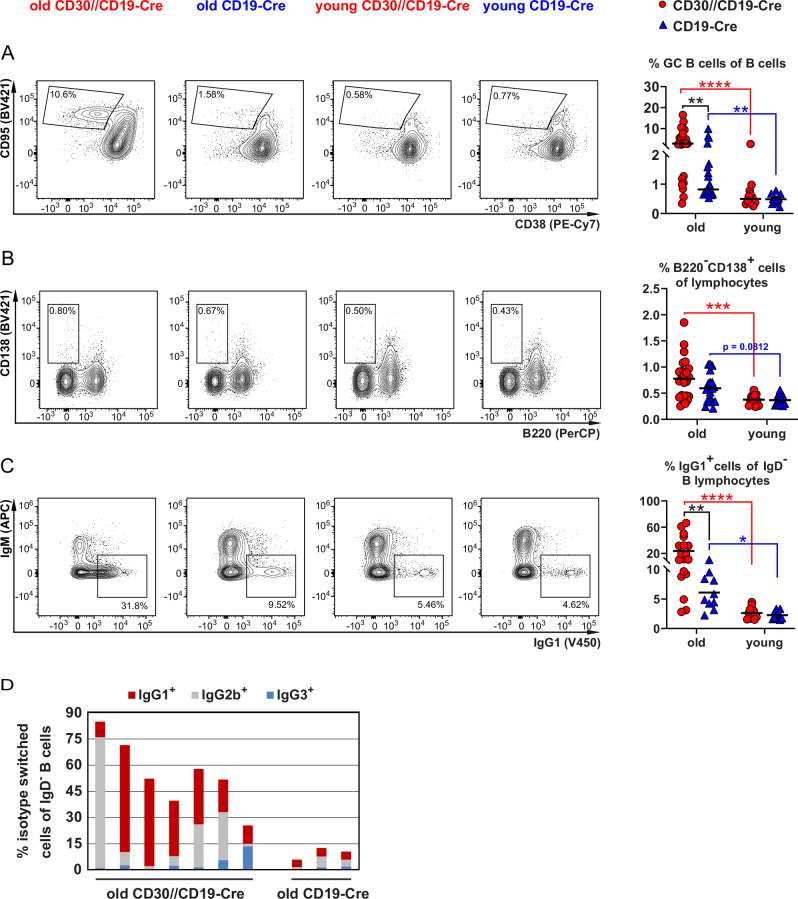
Compared with control mice, aged CD30//CD19-Cre mice have more GC B cells and more IgG1-switched cells. **A** FACS plots are pregated on living B220^+^ lymphocytes, as shown in Supplementary Fig. 5a. GC B cells were gated as CD95^high^CD38^−^ cells, as indicated in the FACS plots. The graph summarizes the percentages of GC B cells in young and aged CD30//CD19-Cre mice (red) and control mice (blue) (*N* = 12–25 per group). **B** FACS plots depicting the gating strategy for plasmablasts/plasma cells (B220^−^CD138^+^) in young and aged CD30//CD19-Cre (red) mice and control mice (blue). The FACS plots are pregated on singlets, live cells and a large lymphocyte gate, as shown in Supplementary Fig. 5b. The percentages of PCs in different analyses are compiled in the accompanying graph (*N* = 11–24). **C** The graph compiles the percentages of isotype-switched IgG1^+^ B cells of the indicated genotypes at the indicated ages (*N* = 10–18 per group). IgG1-switched B cells were gated as single living CD19^+^IgD^−^ lymphocytes as indicated in Supplementary Fig. 5a and b. **D** The graph shows the percentages of isotype- switched IgG1 (red), IgG2b (gray) and IgG3 (blue) cells in aged CD30//CD19-Cre mice and aged control mice. **A**–**C** Data from 18 flow cytometric analyses. Two-way ANOVA with Tukey’s multiple comparisons test. *P ≤ 0.05, **P ≤ 0.01, ***P ≤ 0.001, ****P ≤ 0.0001

Physiologically, CD30 is expressed in a severely limited number of GC B and post-GC B cells [10]. To investigate whether the expression of CD30 on all B cells influences GC formation, we compared the percentages of GC B cells in CD30//CD19-Cre and control mice. We observed that the percentages of spontaneously formed GC B cells increased during aging, and the increase was significantly greater in CD30//CD19-Cre mice than in control mice, with medians of 2.81% and 0.82% of total B lymphocytes, respectively (Fig. 3A). Additionally, we detected a slight but discernable increase in the percentage of plasma cells in the CD30//CD19-Cre mice during aging (Fig. 3B). Compared with old control mice, aged CD30//CD19-Cre mice presented strongly increased percentages of isotype-switched B cells. In most cases, the cells had switched to IgG1; however, increased class switching to IgG2b and to IgG3 was also observed (Fig. 3C, D).

### CD30 expression on B cells results in the expansion of CD30-L-expressing CD44^+^PD-1^+^ SA-T cells and CD30-L-expressing CD44^+^Bcl6^+^ Tfh cells

**Fig. 4:**
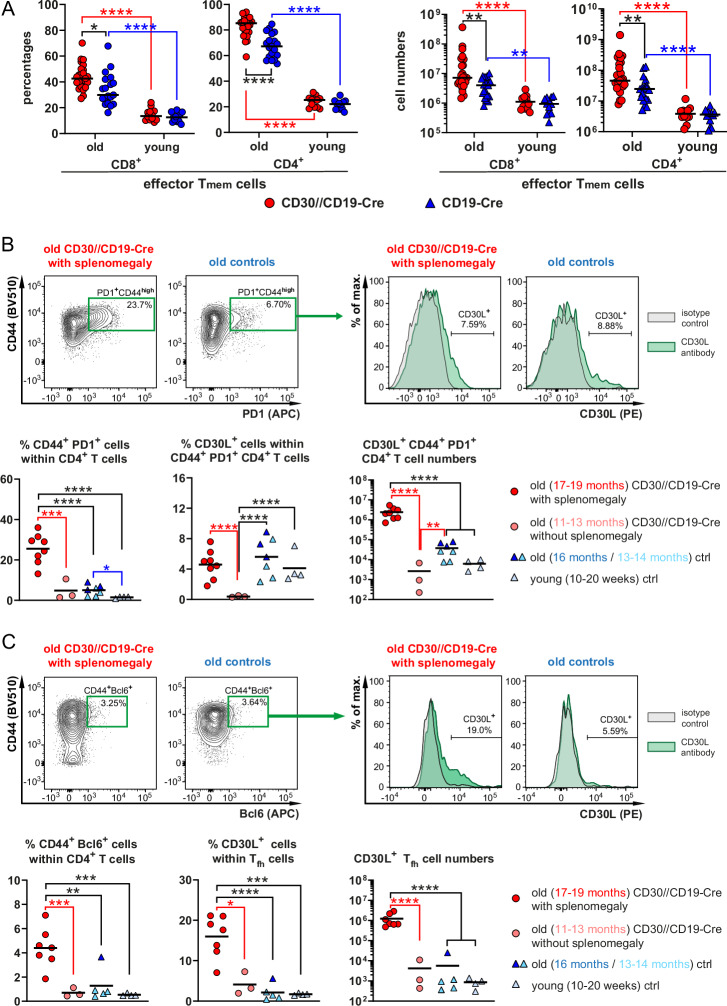
Activated CD4+ T cells express CD30-L **A** Graphs summarizing the percentages of effector memory T cells in the CD4^+^ and CD8^+^ subsets in young and aged CD30//CD19-Cre mice (red) and control mice (blue). Effector memory CD4^+^ and CD8^+^ T cells were gated as CD62L^low^CD44^+^ cells, as indicated in Supplementary Fig. 6a (arrow 1) (*N* = 16–25 per group). Data from 18 flow cytometric analyses. **B** SA-T cells were gated as CD4^+^CD44^high^PD-1^+^ T cells. FACS plots were pregated on singlets and live cells. The plots were subsequently gated on lymphocytes, Thy1.2^+^ cells and CD4^+^ cells, as shown in Supplementary Fig. 6a (arrow 2). The surface expression of CD30-L (CD153) in the gate of SA-T cells is depicted in histograms, in which the stainings with the anti-CD153 antibody (green) and the corresponding isotype control (gray) are overlaid. The graphs compile percentages of CD4^+^CD44^+^PD-1^+^ T cells (left graph), percentages of CD30-L-expressing T cells within CD4^+^CD44^+^PD-1^+^ T cells (middle graph) and total numbers of CD153^+^CD4^+^CD44^+^PD-1^+^ T cells from CD30//CD19-Cre mice (red) and controls (CD19-Cre^+/-^ or CD30^stopfl/+^) (blue) (*N* = 3–8 mice per group). **C** Tfh cells were gated as CD4^+^CD44^+^Bcl6^+^ cells as indicated in the FACS plot. The FACS plots were pregated in the same way as described for the SA-T cells in (**B**). The surface expression of CD30-L (CD153) in the gate of Tfh cells (Thy1.2^+^CD4^+^CD44^+^Bcl6^+^) is depicted in the histograms, which show an overlay of the CD153 staining and the corresponding isotype control. The graphs compile percentages of CD4^+^CD44^+^Bcl6^+^ T cells (left graph), percentages of CD30-L-expressing T cells within CD4^+^CD44^+^Bcl6^+^ T cells (middle graph) and total numbers of CD153^+^CD4^+^CD44^+^Bcl6^+^ T cells (right graph) from CD30//CD19-Cre mice (red) and controls (CD19-Cre^+/–^ or CD30^stopfl/+^) (blue) (*N* = 3–7 mice per group). The ages of the mice in the different groups from (**B**) and (**C**) are indicated in the figure legends. **A** 2-way ANOVA with Tukey’s multiple comparisons test. **B** + **C**, ordinary one-way ANOVA with Tukey’s multiple comparisons test. *P ≤ 0.05, **P ≤ 0.01, ***P ≤ 0.001, ****P ≤ 0.0001

Young CD30//CD19-Cre mice exhibit the same B-cell phenotype as control mice do, but with aging, they show enhanced B-cell and T-cell expansion, spontaneous GC formation and increased class-switch activity. These findings suggest that CD30-expressing B cells are predominantly stimulated in aged mice. As shown in Fig. 2B, total T-cell numbers increased with age, particularly in CD30//CD19-Cre mice, with a shift from naïve T cells to effector memory T cells (Fig. 4A, Supplementary Fig. 6a+b). This finding suggests reciprocal stimulation between CD30-expressing B cells and CD30-L-expressing T cells. Recent studies have characterized the expansion of senescence-associated T (SA-T) cells as CD4^+^CD44^high^PD-1^+^ cells during aging [20]. Indeed, we observed significant expansion of these SA-T cells in 17–19-month-old CD30//CD19-Cre (Fig. 4B, left graph) compared with young and old control mice. A certain percentage of these SA-T cells expressed CD30-L. The percentages of CD30-L-expressing cells within SA-T cells were comparable between 17–19-month-old CD30//CD19-Cre and aged control mice but were significantly lower in 14-month-old CD30//CD19-Cre mice, suggesting the counterregulation of CD30-L-expressing T cells by the abundance of CD30^+^ B cells (Fig. 4B, middle graph). The total number of CD30-L-expressing SA-T cells was significantly greater in 17–19-month-old CD30//CD19-Cre mice than in old (53-fold) and young (325-fold) control mice and middle-aged (756-fold) CD30//CD19-Cre mice (Fig. 4B, right graph). In 17–19-month-old CD30//CD19-Cre mice, SA-T-cell expansion was accompanied by pronounced splenomegaly, which was not yet visible in 14-month-old CD30//CD19-Cre mice (Supplementary Fig. 6c). Additionally, Tfh cells expressing CD30-L were significantly expanded in 17–19-month-old CD30//CD19-Cre mice (Fig. 4C). These results indicate that aging leads to a marked increase in the number of CD30-L-expressing T cells, particularly SA-T cells and Tfh cells, in CD30//CD19-Cre mice, which in turn stimulates a greater number of B cells, resulting in predominant phenotypes in aged mice.

### CD30 signaling results in more germinal center B cells at early time points after immunization

**Fig. 5:**
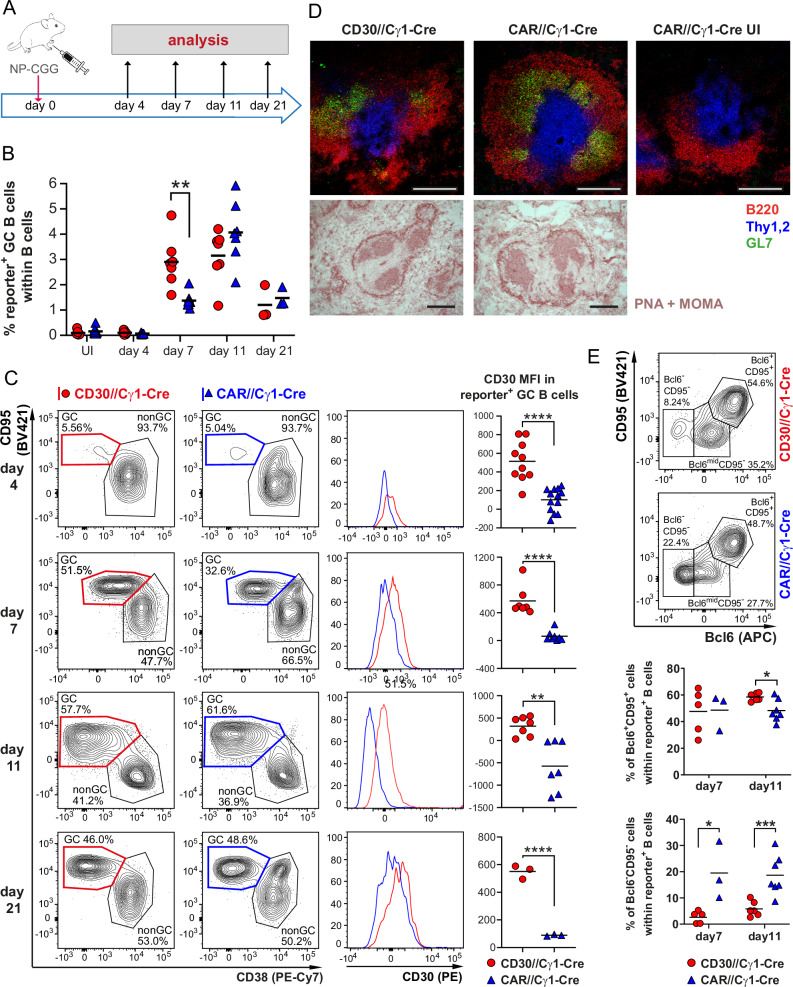
GC kinetics are accelerated in transgenic CD30 mice. **A** Treatment scheme: CD30//Cγ1-Cre mice were immunized with NP-CGG and analyzed at the indicated time points. **B** The graph summarizes the percentages of reporter^+^ GC B cells at different time points after immunization. UI: unimmunized controls; red dots represent CD30//Cγ1-Cre mice, and blue triangles represent CAR//Cγ1-Cre mice (controls). Reporter^+^ cells were gated as described in Supplementary Fig. 7a (*N* = 3–13 per group). **C** CD30 expression is increased in GC B cells from CD30//Cγ1-Cre mice (red dots) compared with controls (CAR//Cγ1-Cre mice; blue triangles). The FACS plots indicate the gating of GC B cells (CD95^+^CD38^−^) at different time points after immunization. The FACS plots were sequentially pregated on living single cells, lymphocytes, B cells (B220^+^) (as shown in Supplementary Fig. 5a) and reporter^+^ cells (Supplementary Fig. 7a). The histogram overlays show the CD30 expression in the GC B cells of the CD30//Cγ1-Cre mice (red lines) versus the controls (CAR//Cγ1-Cre mice; blue lines). The graphs on the right side summarize the median fluorescence intensities (MFIs) of CD30 in GC B cells at the indicated time points after immunization (*N* = 7–13 per group). **B** + **C** Rounds of immunization: day 4: 5; day 7: 5; day 11: 4; day 21: 2. **D** Images of GC B cells detected via immunohistofluorescence (upper row) or immunohistochemistry (lower row) of splenic sections 11 days after immunization. For immunofluorescence staining, B cells were stained with anti-B220 (red), T cells with anti-Thy1.2 (blue) and GC B cells with anti-GL-7 (green). In immunohistochemical staining, metallophilic macrophages lining the marginal zone sinus are stained with anti-Moma-1, and germinal center B cells are stained with PNA; both stains are red in color. The data are representative of 2 immunization rounds. **E** FACS plots demonstrating the gating strategy for Bcl6^+^CD95^high^ (pre-GC and GC B cells) and Bcl6^−^CD95^−^ non-GC B cells. The FACS plots were sequentially pregated on single cells, living cells, lymphocytes, B220^+^ cells and reporter^+^ cells (Supplementary Figs. 5a and 7a). The graphs below summarize the percentages of Bcl6^+^CD95^+^ GC B cells and Bcl6^−^CD95^−^ non-GC B cells at the indicated time points after immunization (*N* = 3–7 per group). Rounds of immunization: day 7: 2; day 11: 3. **B** 2-way ANOVA with Sidak’s multiple comparisons test. **C** + **E**, Unpaired *t* tests, *P ≤ 0.05, **P ≤ 0.01, ***P ≤ 0.001, ****P ≤ 0.0001

The influence of CD30 signaling on B-cell expansion during the aging of CD30//CD19-Cre mice was notably observed in GC B cells, IgG1-switched B cells, and CD43^+^CD23^–^ cells, most likely consisting of antigen-activated B cells [14, 21]. These findings strongly imply that the expansion of CD30-expressing B cells physiologically occurs during antigen stimulation. To investigate the effects of CD30 signaling on immune responses in more detail, we crossed CD30^stopfl/+^ mice with the Cγ1-Cre strain [22] (CD30//Cγ1-Cre mice), in which the stop cassette was deleted upon TD immunization. For the control mice, we used the reporter strain R26-CARΔ1^stopF/+^ [23], which contains the coding sequence for the coxsackie/adenovirus receptor (CAR) at the *Rosa26* locus in combination with a loxP-flanked stop cassette (CAR//Cγ1-Cre hereafter). Immunization with the TD antigen NP-CGG leads to the expression of CD30 and the reporter hCD2 on the surface of GC B and post-GC B cells in CD30//Cγ1-Cre mice and to the expression of the reporter CAR in the respective cells in CAR//Cγ1-Cre mice. The mice were analyzed at different time points after immunization (p.i.) (Fig. 5A). CD30//Cγ1-Cre mice presented a more rapid increase in reporter^+^ cells, peaking at ~7% on day 7. Afterwards, the percentages of reporter^+^ cells remained stable until day 11 (Supplementary Fig. 7a). In contrast, CAR//Cγ1-Cre mice presented a more gradual increase in the percentage of reporter^+^ cells until day 11, when they reached percentages similar to those of CD30//Cγ1-Cre mice. Both CD30//Cγ1-Cre and CAR//Cγ1-Cre mice subsequently presented decreases in the proportions of reporter^+^ B cells. In both genotypes, total and reporter^+^ GC B cells increased and decreased, respectively, with the same kinetics as reporter^+^ cells (Fig. 5B, Supplementary Fig. 7b), indicating that GCs are established faster in CD30//Cγ1-Cre mice than in controls in the first week of the immune response (day 7). CD30 expression was significantly greater in the reporter^+^ GC B cells of the CD30//Cγ1-Cre mice than in those of the control mice at all the analyzed time points postimmunization (Fig. 5C). The GC structures were well organized in both the mutant and control mice (Fig. 5D). The percentage of reporter^+^Bcl6^–^CD95^–^ B cells was significantly lower in the CD30//Cγ1-Cre mice than in the control mice, whereas the percentage of Bcl6^+^CD95^+^ B cells was significantly greater by day 11 postimmunization, further strengthening our assumption that more B cells become activated and enter the GCs in the CD30//Cγ1-Cre mice during the immune response (Fig. 5E). These data suggest that CD30 signaling facilitates faster entry of pre-GC cells into GCs.

### CXCR4 surface levels are increased in DZ GC derived B cells from CD30//Cγ1-Cre mice

**Fig. 6:**
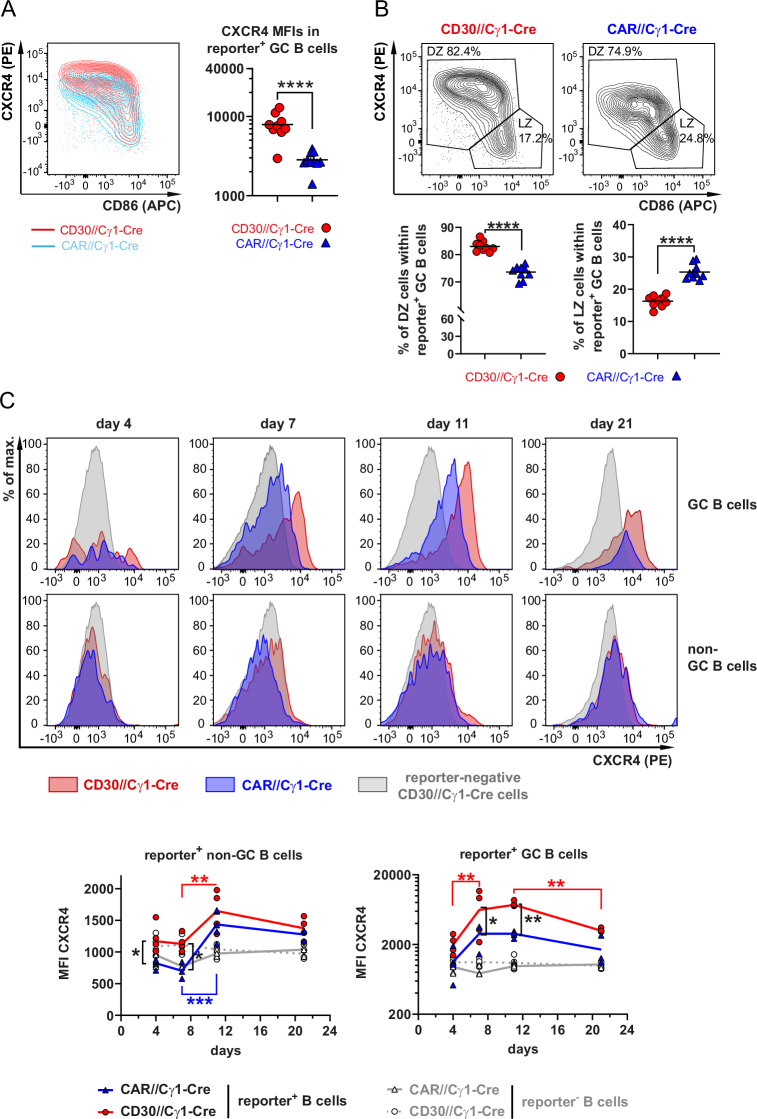
CXCR4 levels are increased in GC B cells from transgenic CD30//Cγ1-Cre mice. All analyses shown in this figure are from the spleen. FACS plots are pregated on singlets, live cells, lymphocytes and B220^+^ cells, as shown in Supplementary Fig. 5a, and on GC B cells, as shown in Fig. 5C. **A** The FACS plot overlay of reporter^+^ GC B cells from CD30//Cγ1-Cre mice (red) and controls (blue) illustrates the upregulation of CXCR4 in GC B cells from CD30//Cγ1-Cre mice. The graph compiles the median fluorescence intensity (MFI) of CXCR4 in GC B cells from both indicated genotypes on day 11 postimmunization (*N* = 9–10 per group). Rounds of immunization: 6. **B** FACS plots showing a representative example of the gating strategy for dark zone (DZ) (CXCR4^high^CD86^low^) and light zone (LZ) (CXCR4^low^CD86^high^) reporter^+^ GC B cells. The graphs below summarize the percentages of DZ and LZ reporter^+^ GC B cells 11 days after immunization (*N* = 9–10 per group). Rounds of immunization: 6. **C** The histogram overlays show the CXCR4 levels in reporter^+^ GC and reporter^+^ non-GC B cells (gated as shown in Fig. 5) in comparison to those in reporter^‒^ cells at the indicated time points postimmunization. The graphs below summarize the MFIs from CXCR4 in reporter^+^ GC B cells (right graph) and reporter^+^ non-GC B cells (left graph) in comparison to those in reporter^‒^ cells (*N* = 3‒5 per group). **A** + **B**, Unpaired *t* test. **C** 2-way ANOVA with Sidak’s multiple comparisons test. *P ≤ 0.05, **P ≤ 0.01, ***P ≤ 0.001, ****P ≤ 0.0001

We observed higher CXCR4 levels on the cell surface of reporter^+^ GC B cells from CD30//Cγ1-Cre mice than in those from control mice (Fig. 6A, B), which affected predominantly DZ GC B cells. High CXCR4 expression guides B cells to the DZ, whereas downregulation of CXCR4 in the DZ allows B cells to migrate to the LZ [24–26]. In line with the higher CXCR4 expression, we observed a greater percentage of DZ GC B cells in the CD30//Cγ1-Cre mice than in the controls (Fig. 6B). The kinetics of CXCR4 upregulation at different time points postimmunization revealed that in reporter^+^ non-GC B cells, differences between mutant and control mice were most evident at early time points after immunization (Fig. 6c, left graph), potentially explaining the accelerated entry of B cells into the GC in the CD30//Cγ1-Cre mice compared with the controls (as shown in Fig. 5B). In reporter^+^ GC B cells, significant differences in surface CXCR4 expression between CD30//Cγ1-Cre and control mice were primarily observed at the peak of the GC reaction at days 7 and 11 (Fig. 6C, right graph). As expected, both genotypes displayed comparably low CXCR4 expression in reporter^‒^ B cells. To explore whether CXCR4 is a transcriptional target gene of CD30 signaling, we investigated CXCR4 mRNA levels in sorted reporter+ GC and reporter^+^ non-GC cells from CD30//Cγ1-Cre mice and control mice 11 days postimmunization via qRT‒PCR (Supplementary Fig. 8a). As expected, *Bcl6* mRNA levels were higher in GC B cells than in non-GC B cells, and *CD30* mRNA was strongly increased in B cells sorted from CD30//Cγ1-cre mice compared with those from controls, validating the accuracy of our internal controls. Unexpectedly, *Cxcr4* mRNA levels were similar between CD30//Cγ1-Cre and control mice in both GC and non-GC B cells (Supplementary Fig. 8b). Additionally, no significant difference in *Cxcr4* mRNA expression was observed between reporter^+^ GC cells and non-GC cells, although GC B cells clearly displayed higher CXCR4 protein levels on the cell surface than non-GC B cells did (Supplementary Fig. 8c). These findings suggest that differences in CXCR4 protein expression between genotypes and cell types are not correlated with mRNA levels, indicating that CXCR4 surface expression is likely regulated primarily at the posttranscriptional rather than the transcriptional level.

### CD30 signaling enhances plasma cell differentiation upon TD immunization

**Fig. 7:**
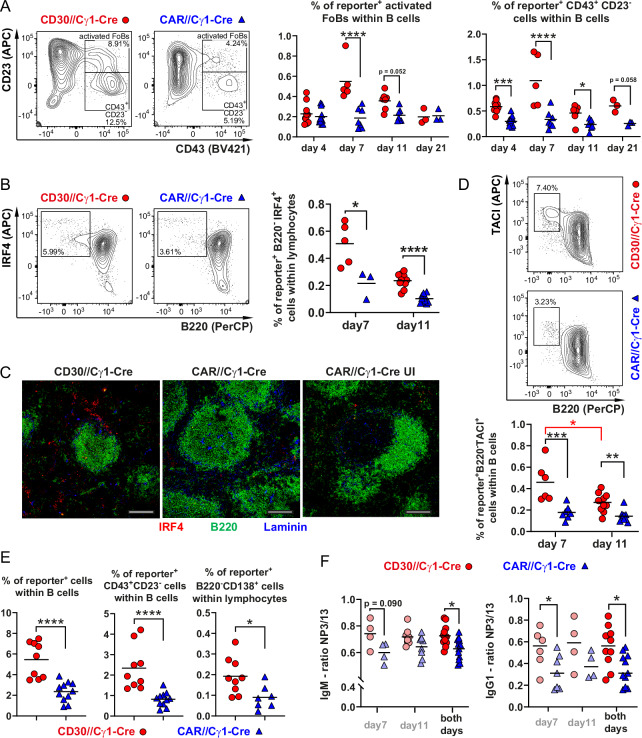
Plasma cell differentiation is enhanced by transgenic CD30 expression. **A** FACS plots illustrating the gating strategy for activated FoB cells (CD43^+^CD23^+^) and plasmablasts + B1 cells (CD43^+^CD23^−^). The FACS plots were pregated as shown in Supplementary Fig. 3a. CD19^+^ cells were subsequently gated on reporter^+^ cells. The graphs summarize the percentages of reporter^+^ activated FoB cells and CD43^+^CD23^−^ B cells in the spleen at the indicated time points after immunization. CD30//Cγ1-Cre mice (red dots), control mice (CAR//Cγ1-Cre mice; blue triangles) (*N* = 3–13 per group). Rounds of immunization: day 4: 5; day 7: 4; day 11: 4; day 21: 2. **B** Gating strategy for plasmablasts (IRF4^+^B220^low^) in the spleen. The FACS plots were pregated as indicated in Supplementary Fig. 5b. The cells in the large lymphocyte gate were subsequently gated on reporter^+^ cells. The graph compiles the percentages of IRF4^+^B220^low^ cells in CD30//Cγ1-Cre mice (red dots) and controls (CAR//Cγ1-Cre mice; blue triangles) on day 7 and day 11 after immunization (*N* = 3–11 per group). Rounds of immunization: day 7: 2; day 11: 7. **C** Immunohistochemistry images depict the localization of IRF4^+^ cells (red) in histological sections from the spleen on day 11 after immunization. B cells were stained with B220 (green), and the marginal sinus was stained with anti-Laminin (blue). **D** FACS plots of the gating strategy for plasma cells (TACI^+^B220^low^) are shown. The FACS plots were pregated as indicated in Supplementary Fig. 5b. The cells in the large lymphocyte gate were subsequently gated on reporter^+^ cells. The graphs summarize the percentages of reporter^+^ plasma cells at days 7 and 11 after immunization (*N* = 6–11 per group). Rounds of immunization: day 7: 4; day 11: 6. **E** The graphs compile the percentages of reporter^+^ B cells (left), reporter^+^ plasmablasts and B1 cells (CD43^+^CD23^−^) (middle), and reporter^+^ plasma cells (CD138^+^B220^low^) (right) in the blood. CD30//Cγ1-Cre mice (red dots) and controls (CAR//Cγ1-Cre mice; blue triangles) are shown (*N* = 9–11 per group). Rounds of immunization: 5. **F** The serum titers of NP-specific antibodies were determined by ELISA using NP3-BSA (for high-affinity NP-specific antibodies) or NP13-BSA (for total NP-specific antibodies) as the capture reagent. The ratios of high (binding to NP3) to total NP-specific (binding to NP13) antibodies for the IgM and IgG1 subclasses were determined on day 7 and day 11 after immunization (*N* = 4–8 per group). In the last row, the ratios of NP3/NP17 from day 7 and day 11 were combined. The relative titers of anti-NP-IgM and anti-NP-IgG1 antibodies bound to NP-13-BSA and NP-3-BSA in the sera of CD30//Cγ1-Cre mice and control mice are shown in Supplementary Fig. 9b. 2-way ANOVA with Sidak’s (**A**) or Tukey’s (**D**) multiple comparisons test. **B** + **F**, Unpaired *t* tests. **E** Unpaired *t* test. *P ≤ 0.05, **P ≤ 0.01, ***P ≤ 0.001, ****P ≤ 0.0001

Recently, we showed that constitutively active CD30 signaling (LMP1/CD30//CD19-Cre mice) in B cells results in the expansion of CD43^+^CD23^–^B220^low^ B cells, likely representing extrafollicular preplasmablasts [14, 21]. As illustrated in Fig. 2F, this population was also elevated in the old CD30//CD19-Cre mice compared with the controls. In accordance with these previous results, we observed a notable increase in CD43^+^ B cells, which were either CD23^+^ or CD23^–^ (Fig. 7A), after TD immunization of CD30//Cγ1-Cre mice. In addition, reporter^+^IRF4^+^B220^low^ B cells (Fig. 7B), which were predominantly located in the extrafollicular region (Fig. 7C), as well as reporter^+^ B220^–^TACI^+^ cells (Fig. 7D), were notably expanded in the CD30//Cγ1-Cre mice compared with the controls. Increased percentages of reporter^+^CD43^+^CD23^−^ cells and reporter^+^B220^–^CD138^+^ plasma cells were also detected in the blood (Fig. 7E). These findings suggest that PC differentiation is enhanced in CD30//Cγ1-Cre mice than in controls. To assess whether the increased percentages of reporter^+^ plasmablasts and plasma cells correlate with higher titers of total and NP-specific antibodies, we performed ELISAs. The total IgM titer was significantly higher in the CD30//Cγ1-Cre mice than in the control mice both in the unimmunized state and at 11 days postimmunization. Additionally, total IgG1 antibodies were significantly elevated at all three time points (unimmunized, day 7, and day 11 postimmunization) (Supplementary Fig. 9a). These results are consistent with our observation of more PCs in CD30//Cγ1-Cre mice than in control mice. Furthermore, we detected higher NP-specific IgM titers with high affinity on day 7 and higher IgG1 titers with high affinity on day 11 postimmunization (Supplementary Fig. 9b). Calculation of the ratio of titers from antibodies binding to NP3 (high-affinity antibodies) or NP13 (total antibodies) revealed that the NP3/NP13 binding ratio for IgM- and IgG1-specific NP-specific antibodies was significantly higher in CD30//Cγ1-Cre mice than in controls (Fig. 7F). These findings suggest that after TD immunization, more NP-specific high-affinity PCs exit the GC in CD30//Cγ1-Cre mice than in controls.

### CD30 signaling increases the percentage of IgG1 isotype-switched cells in the GC

**Fig. 8:**
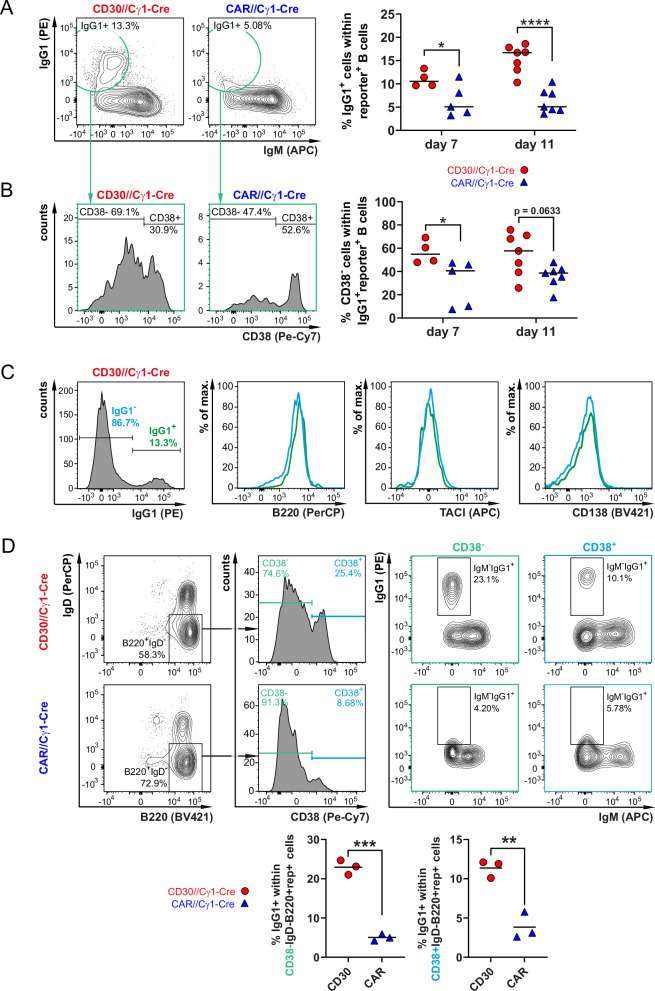
CD30 signaling in GC B cells induces the expansion of IgG1-switched cells. All analyses were performed with B cells from the spleen. **A** FACS plots showing the exemplary gating of IgG1-switched cells in CD30//Cγ1-Cre mice and controls. The FACS plots were pregated on B220^+^ single live lymphocytes, as shown in Supplementary Fig. 5a. B220^+^ cells were subsequently gated on reporter^+^ cells. The graph summarizes the percentages of IgG1^+^ cells in CD30//Cγ1-Cre mice (red dots) and controls (CAR//Cγ1-Cre mice; blue triangles) at the indicated time points postimmunization (p.i.) (*N* = 4–7 per group). **B** The histograms depict an exemplary gating strategy of IgG1^+^CD38^+^ non-GC and IgG1^+^CD38^−^ GC B cells. The histograms were pregated as described in (**A**). The graph on the right shows the percentages of CD38^−^ GC B cells within the fraction of IgG1^+^reporter^+^ B cells at the indicated time points p.i. (*N* = 4–7 per group). **A** + **B**, Rounds of immunization: day 7: 3; day 11: 4. **C** To explore the differentiation of IgG1^+^ cells toward plasma cells, the cells were pregated as live large lymphocytes (Supplementary Fig. 5b) and reporter^+^ IgG1^+^ cells. The B220, TACI, and CD138 expression, which are downregulated (B220) or upregulated (TACI and CD138) during PC differentiation, was subsequently analyzed in the cells. The single gray histogram shows the exemplary gating of IgG1^+^ and IgG1^−^ reporter^+^ lymphocytes in CD30//Cγ1-Cre mice. The histogram overlays depict the expression of the indicated markers in IgG1^+^ and IgG1^−^ reporter^+^ B cells. The analysis was performed 11 days after immunization. Data are representative of 6 flow cytometric analyses. **D** FACS plots are sequentially gated on single cells, live lymphocytes, B220^+^ B cells (Supplementary Fig. 5a), and reporter^+^ B cells. To investigate the potential memory B-cell phenotype of IgG1-type-switched B cells, reporter^+^ B cells (B220^+^) were gated on IgD^−^ cells and subsequently subdivided into CD38^+^ (memory B cells) and CD38^−^ (GC B cells). The graphs compile the percentages of IgG1-switched cells within the CD38^+^ and CD38^−^ fractions of reporter^+^B220^+^IgD^−^ cells in CD30//Cγ1-Cre mice (red dots) and controls (CAR//Cγ1-Cre mice; blue triangles) from day 11 postimmunization (p.i.) (*N* = 3 per group). Data from 2 flow cytometric analyses. **A** + **B**, 2-way ANOVA with Sidak’s multiple comparisons test. **D** Unpaired *t* test. *P ≤ 0.05, **P ≤ 0.01, ***P ≤ 0.001, ****P ≤ 0.0001

Weniger and colleagues reported that ~50% of CD30^+^ B cells are IgG1 isotype-switched [10]. This led us to explore whether CD30 signaling influences the amount of IgG1 isotype-switched cells. Indeed, we detected a greater percentage of IgG1^+^ cells in CD30//Cγ1-Cre mice (Fig. 8A), most of which were CD38^–^ B cells, suggesting a GC phenotype (Fig. 8B). Compared with IgG1^‒^ cells, IgG1^+^ cells did not show an increase in CD138 or TACI or a decrease in B220, suggesting that these cells do not differentiate into plasma cells (Fig. 8C). To determine whether the IgG1^+^ cells in the CD30//Cγ1-Cre mice presented a memory-like phenotype, we gated them on reporter^+^B220^+^IgD^−^CD38^+^ cells and determined the percentage of IgG1^+^ cells. In CD30//Cγ1-Cre mice, we detected an IgG1^high^ population that was absent in controls (Fig. 8D). IgG1^high^ cells were also detected within the population of reporter^+^B220^+^IgD^−^CD38^−^ B cells, suggesting that the IgG1^+^ population contains a mixture of GC and post-GC B cells. We postulate that this reporter^+^ IgG1^high^ cell population is generated in the GC (CD38^−^) in response to strong CD30 signaling and subsequently leaves the GC as memory-like B cells (CD38^+^), which, in contrast to control B cells, express high levels of IgG1 on their cell surface. We assume that the expanded CD38^+^ and CD38^–^ IgG1^+^ cells, which we observed in CD30//Cγ1-Cre mice, correspond to the human CD30^+^ GC and non-GC cells that were further characterized by the Küpper´s group [10]. Our findings demonstrate that CD30 signaling actively contributes to the differentiation of these cells in GC and/or pre-GC B cells.

## Discussion

In this study, we provide new insights into the previously elusive role of CD30 signaling in B cells. By utilizing a novel conditional CD30-knockin mouse strain, we revealed compelling evidence that CD30 expression in all B cells (CD30//CD19-Cre mice) leads to notable B-cell expansion in aged mice, with elevated percentages of CD23^low^CD43^+^ cells, GC B cells, and IgG1 isotype-switched cells compared with those in the control group. Our analysis involving mice in which CD30 expression is induced specifically in GC B cells upon TD immunization highlights the functional importance of CD30 signaling in influencing GC dynamics, PC differentiation, and the formation and/or expansion of IgG1 isotype-switched cells. The latter most likely leave the GC as memory-like B cells, characterized by strong IgG1 expression.

We are aware that the results obtained with a conditional knock-in mouse line must be critically interpreted. The overexpression of a transgene could produce artificial results. However, we can show that the surface expression of CD30 in CD30//CD19-Cre and CD30//Cγ1-Cre mice is within a physiological range, suggesting that it can still be regulated at the posttranscriptional level (Fig. 1E, low increase in FoBs and MZBs, comparable bimodal expression in the CD21^low^/CD23^low^ fraction of knock-in and control mice). In addition, the use of the Cγ1-Cre line restricted the expression of CD30 on B cells, which are involved in the immune response. Recent publications [27–29] have confirmed that CD30 is an important member of the TNFR superfamily with respect to B/T-cell interactions during the immunological response process. Nevertheless, conditional CD30-knockout mice are included in our experimental pipeline to confirm and complete our existing results.

In CD30//CD19-Cre mice, B-cell expansion predominantly affects FoB cells and CD43^+^CD23^low^ cells, indicating that antigen-activated B2 cells adopt a preplasmablastic phenotype. Most aged CD30//CD19-Cre mice developed strong splenomegaly, which was caused by the expansion of B cells, T cells and myeloid cells. We have recently shown that constitutively active CD30 signaling in B cells leads to a similar phenotype [14]; however, B cells with constitutively active CD30 expanded more rapidly than those expressing ligand-dependent CD30. This finding suggests that stimulation by CD30-ligand (CD30-L)-expressing cells must precede B-cell expansion in CD30//CD19-Cre mice. Since young and middle-aged CD30//CD19-Cre mice exhibit phenotypes similar to those of control mice, we assumed that extensive stimulation of CD30-expressing B cells primarily occurs in aged mice. Recent studies revealed that CD4^+^CD44^high^PD-1^+^ senescence-associated (SA-T) T cells as well as Tfh cells expand during aging, with some of these T cells expressing CD30-L [27–29]. Notably, we observed greater expansion of CD30-L-expressing SA-T cells and Tfh cells in aged CD30//CD19-Cre mice than in aged control mice. SA-T cells are typically resistant to TCR stimulation. However, interactions with CD30^+^ B cells lead to the association of CD30-L with the TCR/CD3 complex overcoming this refractory state and leading to pathological activation and proliferation of SA-T cells [29]. The greater number of CD30^+^ B cells in these mice likely increased CD30/CD30-L interactions between CD30^+^ B and CD30-L^+^ SA-T cells, enhancing reciprocal stimulation, which results in expansion of both populations. This interaction may also increase cytokine and chemokine production, recruiting myeloid cells. Notably, splenomegaly develops only in very old mice, likely due to strong counterregulation of CD30/CD30-L interactions in young and middle-aged CD30//CD19-Cre mice, where CD30 levels on the cell surface of B cells are low (as shown in Fig. 2D) and the expansion of CD30-L-expressing T cells is limited (Fig. 4B).

In both humans and mice, the expression of CD30 is physiologically tightly regulated [11, 30], suggesting that CD30 signaling is selectively induced within a narrow window of B-cell differentiation. There are a few exceptions in which elevated numbers of CD30-expressing B cells are observed in humans. These include some viral infections, for instance with Epstein–Barr virus, and some autoimmune diseases, such as systemic lupus erythematosus (SLE), rheumatoid arthritis (RA) and Sjögren’s syndrome (SS) [31–33]. Interestingly, in SLE patients, the number of senescence-associated CD4^+^ T cells, which express CD30-L, is significantly increased, and the expansion of these cells is positively correlated with the progression of the disease [34]. Additionally, B-cell expansion and spontaneous ectopic GCs have been detected in RA and SS [35–37], which may be directly linked to enhanced CD30/CD30-L interactions [27, 28, 38].

In the germinal center, CD30 may be selectively upregulated in GC B cells with high-affinity BCRs. These B cells capture, process, and present more antigens than do B cells with a low-affinity BCR, resulting in prolonged interactions with Tfh cells [39–41]. CD30 is normally expressed by only a small subset of GC B cells, so we acknowledge that expressing CD30 in all GC B cells creates an artificial situation. However, the phenotype of these mice aligns with expression data from CD30^+^ GC and non-GC B cells, which suggests that CD30^+^ GC B cells are positively selected centrocytes that return to the DZ [10]. This finding is consistent with our finding that the percentage of DZ B cells is greater in CD30//Cγ1-Cre mice than in controls. The accumulation of CD30-expressing cells in the DZ may affect the affinity maturation of the BCR [26, 42, 43]. The greater ratio of NP3/NP17-binding IgG1 antibodies on day 11 postimmunization provides evidence that affinity maturation is faster in CD30//Cγ1-Cre mice than in control mice.

Moreover, CD30^+^ GC and non-GC B cells are frequently isotype-switched to IgG1 [10]. Our investigation revealed a substantial increase in IgG1^+^ B cells upon immunization of CD30//Cγ1-Cre mice, which included both GC and non-GC B cells. Interestingly, IgG1^+^ cells presented no signs of PC differentiation, suggesting a trajectory toward memory B-cell differentiation. Notably, IgG1^+^ cells in CD30//Cγ1-Cre mice displayed exceptionally high IgG1 expression, possibly a characteristic feature of CD30-expressing cells. Potentially, these CD30^+^ non-GC B cells can be easily stimulated to produce antibodies upon re-encountering antigens. The observed slightly elevated high-affinity IgG1 titers 11 days postimmunization align with our assumption. Under physiological conditions, CD30^+^IgG1-switched non-GC B cells may have a limited lifespan before they undergo apoptosis or lose their activated state and downregulate CD30. The impeded downregulation of CD30 in transgenic CD30 mice may lead to continuous stimulation by CD30-L-expressing cells, ultimately resulting in their accumulation.

Compared with those from control mice, GC B cells from CD30//Cγ1-Cre mice expressed significantly higher levels of CXCR4 on the cell surface, suggesting that CD30 signaling plays a role in upregulating CXCR4 in GC B cells. Our observation aligns with a previous study demonstrating CXCR4 upregulation upon in vitro CD30 stimulation of a CD30^+^ Hodgkin-Lymphoma cell line [44]. Moreover, CXCR4 expression is notably increased on B cells from SLE patients [45], which are suggested to be influenced by CD30 signaling [27–29]. Our data suggest that in GC B cells, CD30 signaling regulates CXCR4 expression primarily posttranscriptionally. Recent studies have shown that CXCR4 signaling can be disrupted by receptor desensitization, a process involving receptor internalization. This desensitization is crucial for restricting extrafollicular immune responses [46]. We propose that CD30 signaling may activate pathways such as the PI3K/Akt and Jak/STAT signaling pathways [10, 14], which inhibit CXCR4 desensitization, leading to increased surface expression. The elevated CXCR4 levels on the cell surface of CD30-expressing B cells could directly contribute to the faster entry of CD30-expressing cells into GCs and to their enhanced differentiation into plasma cells.

In summary, our findings suggest that CD30 signaling plays a pivotal role in orchestrating the fate of GC B cells. We believe that our new findings increase our understanding of the functional role of CD30 signaling during immune responses. Moreover, our discoveries have the potential to shed light on the pathological mechanisms underlying human diseases, which are characterized by increased numbers of CD30^+^ B cells, such as certain autoimmune diseases. Our results imply that diseases marked by a chronically increased population of CD30^+^ B cells should be closely monitored to avoid unspecific B-cell expansions arising from aberrant uncontrolled CD30 signaling.

## Methods

### Mice

Our study examined male and female animals. The data were analyzed without consideration of the sex of the animals. The control and experimental mice were age*-* and sex-matched whenever possible. The experiments were conducted in compliance with the German Animal Welfare Act and were authorized by the institutional committee on animal experimentation and the Government of Upper Bavaria.

To generate CD30^stopfl^ mice, the cDNA of *CD30* [19] was inserted into the *Rosa26* locus via homologous recombination in embryonic stem (ES) cells. The *CD30* transgene is under the control of the CAG promoter and a loxP flanked transcriptional/translational stop cassette, enabling cell type- and tissue-specific expression of the transgene via different Cre strains. At the 3′ priming end of the *CD30* gene, a truncated form (without a signaling tail) of human *hCD2* was inserted as a reporter gene, which is controlled by an internal ribosomal binding site (IRES), resulting in concomitant expression of CD30 and hCD2. To establish the mouse strain CD30^stopfl^, recombinant ES cells were injected into blastocytes. The resulting chimeras were backcrossed with BALB/c wild-type mice to establish the line. For expression of CD30 in all B cells, CD30^stopfl^ mice were mated with CD19-Cre mice [47], and for germinal center-specific expression, Cγ1-Cre mice [22] were used, resulting in CD30^stopfl/+^//CD19-Cre^+/–^ mice (CD30//CD19-Cre) and CD30^stopfl/+^//Cγ1-Cre^+/–^ mice (CD30//Cγ1-Cre), respectively. As controls, we used CD19-Cre^+/–^ (CD19-Cre) [47] for CD30//CD19-Cre mice and R26/CAG-CAR∆1^StopF/+^//Cγ1-Cre for CD30//Cγ1-Cre mice. The reporter strain R26/CAG-CAR∆1^StopF^ was generated and characterized by the Marc Schmidt-Supprian laboratory [23]. The mouse strain contains the human coxsackie/adenovirus receptor CAR at the *Rosa26* locus, preceded by a loxP-flanked STOP cassette (CAR//Cγ1-Cre). TD immunization leads to the simultaneous expression of CD30 and hCD2 in CD30//Cγ1-Cre mice and of CAR in CAR//Cγ1-Cre mice. CD19-Cre and Cγ1-Cre mice were kindly provided by Klaus Rajewsky, MDC Berlin, and R26/CAG-CAR∆1^StopF^ mice were provided by Marc Schmidt-Supprian, TU Munich. All mouse lines were on a BALB/c background and housed in specific-pathogen-free environments.

### Antibodies used in this study

All details about the different antibodies used in flow cytometry (FACS), enzyme-linked immunosorbent assay (ELISA), and histology are listed in Supplementary Table 1.

### Flow cytometry (FACS)

For surface staining, single-cell suspensions of the spleen were prepared, washed and adjusted to a concentration of 5 × 10^6^ cells/ml in MACS buffer (Miltenyi Biotech) on ice. For each round of staining, 5 × 10^5^ cells were used. To identify living cells, the cells were stained with a LIVE/DEAD Fixable Blue Dead Cell Stain Kit (Invitrogen) for 20 min in the dark on ice, followed by surface antibody staining for 20 min in the dark on ice. For intracellular FACS staining, the cells were first stained with the LIVE/DEAD Fixable Blue Dead Cell Stain Kit (Invitrogen) for 5 min on ice, fixed with 2% paraformaldehyde for 10 min at room temperature, and permeabilized in precooled methanol for 10 min on ice. The cells were then stained with antibodies for 1 h at room temperature. For staining with CD30-L and the respective isotype control, mouse FcR blocking reagent (Miltenyi) was added at the appropriate concentration according to the manufacturer´s protocol. Cytometry analysis was performed on an LSRII FACS Fortessa (BD Biosciences) coupled to BD FACS DIVA Software V8.0.1. The results were evaluated via FlowJo (v9 and v10).

### Western blot

MACS-purified splenic B cells were lysed in NP40 lysis buffer (150 mM NaCl; 50 mM Tris/HCl, pH 8; 1% Ipegal), separated on a 10% polyacrylamide SDS gel and transferred to a polyvinylidene fluoride (PVDF) membrane (Immobilon^TM^P membrane). The membrane was stained with the anti-CD30 antibody sc-46683 (Santa Cruz).

### Mouse immunizations

To study T-cell-dependent immune responses, 8- to 16-week-old mice were injected intraperitoneally with 100 µg of alum-precipitated 4-hydroxy-3-nitrophenylacetyl-chicken-gamma-globulin (NP-CGG) (Biosearch Technologies, Novato, CA) in 200 µl of sterile PBS. The immune response of the mice was analyzed at the indicated time points postimmunization.

### ELISA

Antigen-specific ELISA and ELISA for total immunoglobulin were performed as described previously [14]. In brief, 96-well plates (Nunc) were coated with NP3-BSA or NP14-BSA (10 mg/ml, LGC Biosearch Technologies) at 4 °C overnight, after which the process was carried out at room temperature. For the determination of total immunoglobulin titers, plates were coated with purified rat anti-mouse IgM (clone II/41, BD Biosciences, Cat# 553435) or purified rat anti-mouse IgG1 (clone A85-3, BD Biosciences, Cat# 553445) instead, both diluted 1:100 in carbonate-bicarbonate buffer (0.2 M, pH 9.5).

To determine the NP-specific IgM antibody titers, the respective plates were washed with PBST (PBS plus 0.05% Tween 20) and blocked with 5% milk in PBS for 2 h. To determine total immunoglobulin titers, plates coated with purified rat anti-mouse IgG1 were washed with PBS and blocked with 1% milk in PBS for 1 h, while plates coated with purified rat anti-mouse IgM were washed and blocked in the same way as described for the plates in which NP-specific IgM antibody titers were determined.

The sera were diluted in PBS with 1% milk (for IgM, 1:10; for IgG1, 1:100; for total immunoglobulin titers, 1:200) and were added to the plates at 1:2 serial dilutions across 8 wells. The plates were incubated for 1 h at room temperature. The plates were washed and incubated with anti-mouse IgM HRP (clone 1B4B1, Southern Biotech, Cat# 1140-05) or anti-mouse IgG1 biotin (clone A85-1, BD Biosciences, Cat# 553441) for 1 h to detect NP-specific and total IgM and IgG1- in the mouse sera, respectively.

NP-specific and total IgM antibodies were detected directly after this incubation. For NP-specific IgG1 antibodies, the plates were washed with PBS and incubated with streptavidin horseradish peroxidase (HRP) avidin D (Vector) diluted in blocking buffer. For total IgG1 antibodies, the plates were washed with PBS and incubated with alkaline phosphatase (AP) streptavidin (Vector, SA-5100) diluted in blocking buffer.

Finally, the plates were developed with substrate buffer (1 tablet of phenylenediamine dihydrochloride (Sigma, P-7288))+ 35 ml of substrate buffer (0.1 M citric acid, 0.1 M Tris supplemented with 21 µl of H_2_O_2_). The plates incubated with alkaline phosphatase streptavidin were developed with 1 tablet of nitrophenylphosphate (Thermo Fisher Scientific, Cat# 34045) per 5 ml of 1x Pierce™ diethanolamine substrate buffer (Thermo Fisher Scientific, Cat# 34064) for 30 min.

The absorbance was determined with an ELISA plate reader (Photometer Sunrise RC, Tecan) at a wavelength of 405 nm, and data were acquired with Infinite F200 PRO i-control software, version 3.37. To facilitate the comparison of independent assays for NP-specific antibody titers, internal standards derived from mouse sera obtained from mice recently immunized with NP-CGG were employed. To compare independent assays in which total immunoglobulin titers were determined, purified mouse IgM (clone G155--228, BD Biosciences, Cat# 553472) or purified mouse IgG1 (clone MOPC-31C, BD Biosciences, Cat# 557273) were used as standards. Calibration curves were constructed from the absorbance values obtained from the standards. These curves served as reference points to calculate the titers in the sera of the CAR//Cγ1-Cre and CD30//Cγ1-Cre animals via Microsoft Excel 365 and GraphPad Prism software. All the NP-specific titers are presented as relative units, and the total titers are presented as concentrations.

### Histology

The tissues were embedded in O.C.T. compound (VWR Chemicals, USA), snap frozen and stored at –20 °C. The samples were sliced to a thickness of 7 µm with a cryostat, mounted on glass slides and dried for 20 min at room temperature before fixation. For immunofluorescence staining, the slides were fixed with 3% Histofix for 10 min, rinsed with PBS, and rehydrated for 5 min in PBS (supplemented with 50 mM NH_4_Cl) for 5 min. Subsequently, blocking was performed in PBS containing 1% BSA and 5% rat serum for 20 min. For detection of GC B cells, the slides were stained with the following antibodies: anti-GL7-FITC (anti-mouse T- and B-cell activation antigen (GL7)) FITC (clone GL7, BD Biosciences, Cat# 553666) diluted in 1% BSA/PBS at 4 °C overnight. The secondary stains used were as follows: anti-Thy1.2-Biotin (Anti-Mouse CD90.2 clone 30-H12 Biotin, BD Biosciences, Cat# 553011). The third stains used were as follows: SA-594 (Thermo Fisher) and anti-B220-APC (Anti-Mouse CD45R/B220 APC, clone RA3-6B2, BD Biosciences, Cat# 553092) in 1% BSA/PBS for 1 h at room temperature. The following stains were performed for the detection of plasmablasts: first, Rat-anti-mouse Irf4 (Anti-Mouse Irf4 purified antibody, clone 3E4, eBioscience, Cat# 14-9858-80) and rabbit anti-mouse Laminin (Anti-Laminin, clone L9393, Sigma‒Aldrich, Cat# L9393) were diluted in 1% BSA/PBS and incubated for 1 h at room temperature. The secondary stains used were as follows: goat anti-rat Alexa Fluor 488 (anti-rat IgG Alexa Fluor 488, Jackson ImmunoResearch, Cat# 112--545--003) and goat anti-rabbit Cy3 (goat anti-rabbit IgG Cyanine Cy3, Jackson ImmunoResearch, Cat# 111--165--003) diluted in 1% BSA/PBS and incubated for 1 h at room temperature. Third, B220 APCs (Anti-Mouse CD45R/B220 APCs (clone RA3-6B2), BD Biosciences, Cat# 553092) were diluted in 1% BSA/PBS and incubated for 2 h at room temperature. Slides were embedded in SlowFade Glass Antifade (Invitrogen) and imaged on a TCS SP5 II confocal microscope (Leica). For chromogenic immunohistochemistry, the sections were incubated for 10 min in ice-cold acetone (–20 °C) and subsequently air dried for 10 min. The sections were rehydrated for 5 min in PBS at room temperature. The tissue sections were blocked for 20 min with 5% goat serum diluted in PBS and 1% BSA. After being washed with PBS, the slides were incubated with PNA-Biotin (B-1075, Vector) and Anti-Moma-1-Biotin (Anti-Metallophilic Macrophages (MOMA-1), clone MOMA-1, Abcam, Cat# ab51814) for 1 h at room temperature.

After a washing step, the slides were incubated with streptavidin-alkaline phosphatase (S-2890; Sigma‒Aldrich) for 1 h at room temperature. Stained samples were developed with an alkaline phosphatase kit (Vector Red™ Alkaline Phosphatase (Red AP) Substrate Kit - LS-J1086 (lsbio.com)) and embedded in Kaiser´s gelatin.

### FACS sorting and qRT‒PCR

To isolate naïve B cells from total splenocytes, a CD43-microbead B-cell isolation kit (Miltenyi, Cat. No. 130-049-801) was used according to the manufacturer´s protocol. A total of 1–4 × 10^7^ enriched naïve B cells were stained with a LIVE/DEAD Fixable nearIR Dead Cell Stain Kit (Thermo Fisher Scientific, Cat. No. L34975) and subsequently with the surface markers B220 PerCP, CD95 BV421, CD38 PeVio77, and hCD2 Fitc/CAR Fitc. The cells were subsequently resuspended in MACS buffer (Miltenyi, Cat. No. 130--091--221) and passed through a cell strainer with a 35 µm mesh size (Falcon Corning, Cat. No. 352235) immediately before cell sorting. After dead cell and doublet exclusion, three populations of B220-positive cells were sorted: reporter^‒^, reporter^+^ GC B cells and reporter^+^ non-GC B cells. After sorting, the cells were resuspended in 1 ml of TRI Reagent (Sigma‒Aldrich, T9424). FACS was performed on a BD FACSAria III via FACSDiva V8.0.1 software.

For qRT‒PCR, RNA was isolated from >4 × 10^5^ FACS-sorted cells, and cDNA synthesis and quantitative PCR were performed as previously described [48]. Target-specific primers were designed via Primer-BLAST. For improved specificity, matching UPL probes were identified, and real-time PCR was performed as described for UPL assays (Roche). Supplementary Table 2 lists all primer and probe combinations. Throughout, primer efficiencies were >1.9.

### Statistics

All the statistical analyses, including tests for distributions and equal variance and calculations of medians, means and standard deviations (SDs), were performed with GraphPad Prism (versions 9.5.1--10.2.3). The data were subjected to normality and lognormality tests. In the case of a lognormal distribution, the data sets were logtransformed prior to statistical analyses. Sample sizes were determined on the basis of previous experiments and preliminary data. No statistical methods were used to predetermine sample sizes. Control and mutant mice were analyzed in parallel and predominantly used in an age-matched manner whenever possible. The experiments were repeated with different individual animals in at least two independent immunization or cell preparation rounds. Sample sizes and the chosen statistical tests are indicated in each figure legend.

## Supplementary information

Supplemental Material
