# Supplementary material for: CD30 influences germinal center B-cell dynamics and the expansion of IgG1-switched B cells
Source: Cell Mol Immunol. 2024 Oct 17;21(12):1410–25. doi: 10.1038/s41423-024-01219-w (PMC11607414; doi:10.1038/s41423-024-01219-w)
Supplement: Supplementary file 1 — Supplemental Material [file 41423_2024_1219_MOESM1_ESM.pdf]

CD30 influences germinal center B cell dynamics and the expansion of IgG1-switched B cells

Yan Wang, Ursula Rambold, Petra Fiedler, Tea Babushku, Claas Tapken, Kai P. Hoefig, Thomas P. Hofer, Heiko Adler, Ali Önder Yildirim, Lothar J. Strobl, Ursula Zimber-Strobl

Supplementary Material

Supplementary Figures 1-9

Supplementary Table 1: **Antibodies**

Supplementary Table 2: **Primers for qRT PCR**

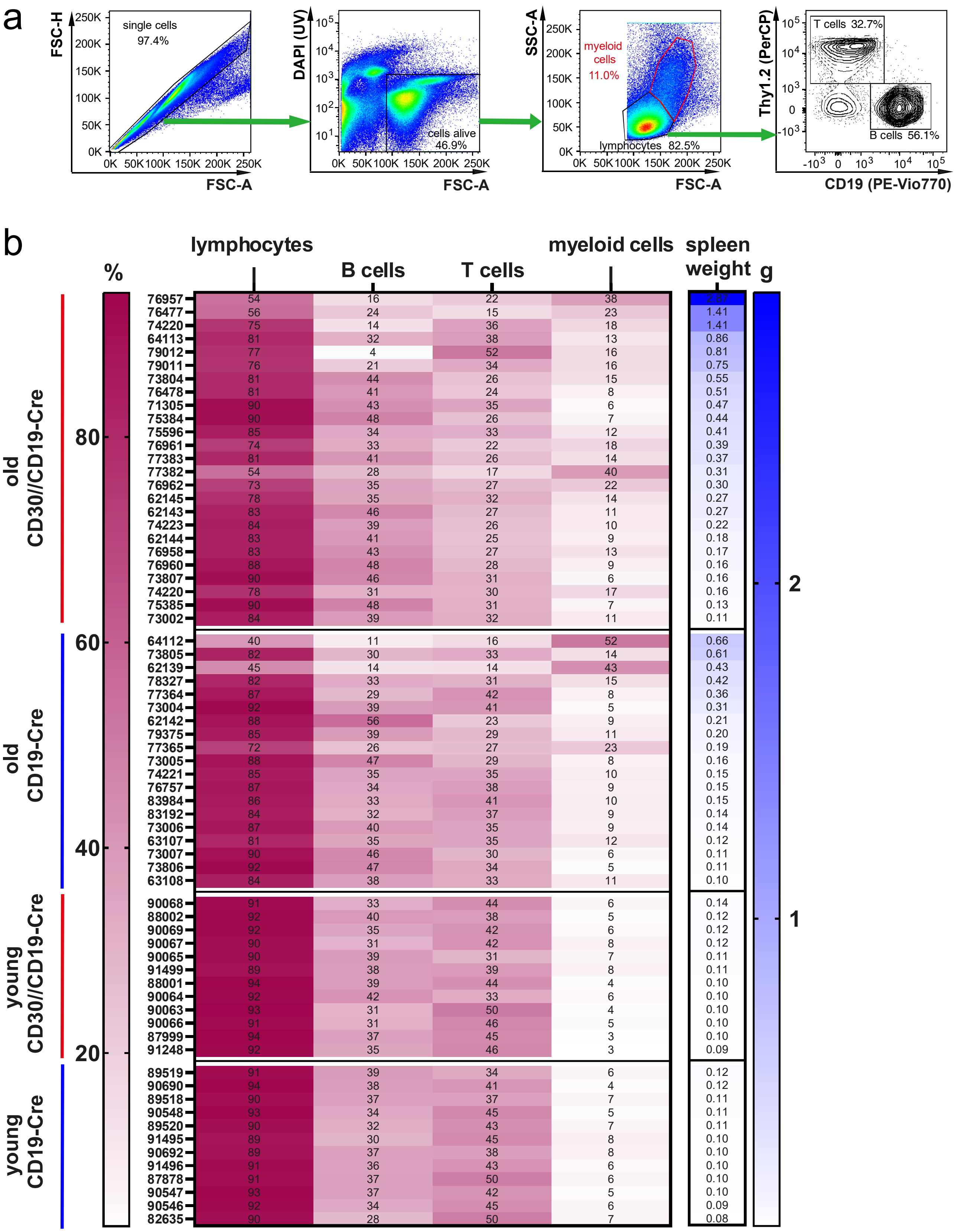

Supplementary Figure 1: Gating strategy for lymphoid and myeloid cells and their distribution in different mice a) gating strategy for data shown in Figure 2b and c. The cells were subsequently gated on singlets and living cells. Living cells were further separated

in the FSC/SSC plot into lymphocytes and myeloid cells as indicated. Within the lymphocyte gate T cells (Thy1.2^{+}) and B cells (CD19^{+}) were identified and gated. **b)** Heat map illustrates the percentage distribution of lymphocytes, B and T cell (as gated in (a)) and myeloid cells (red gate in (a)) within the four mouse cohorts (age groups, genotypes and mouse IDs are indicated). The spleen weight (blue) of individual mice in grams is listed in the right column. The percentages of B and T cells were recalculated based on all spleen cells. The gating strategy is shown in (a). The heatmap compares young CD19-Cre mice, young CD30//CD19-Cre mice, old CD19-Cre mice and old CD30//CD19-Cre mice (N=12-25 per group).

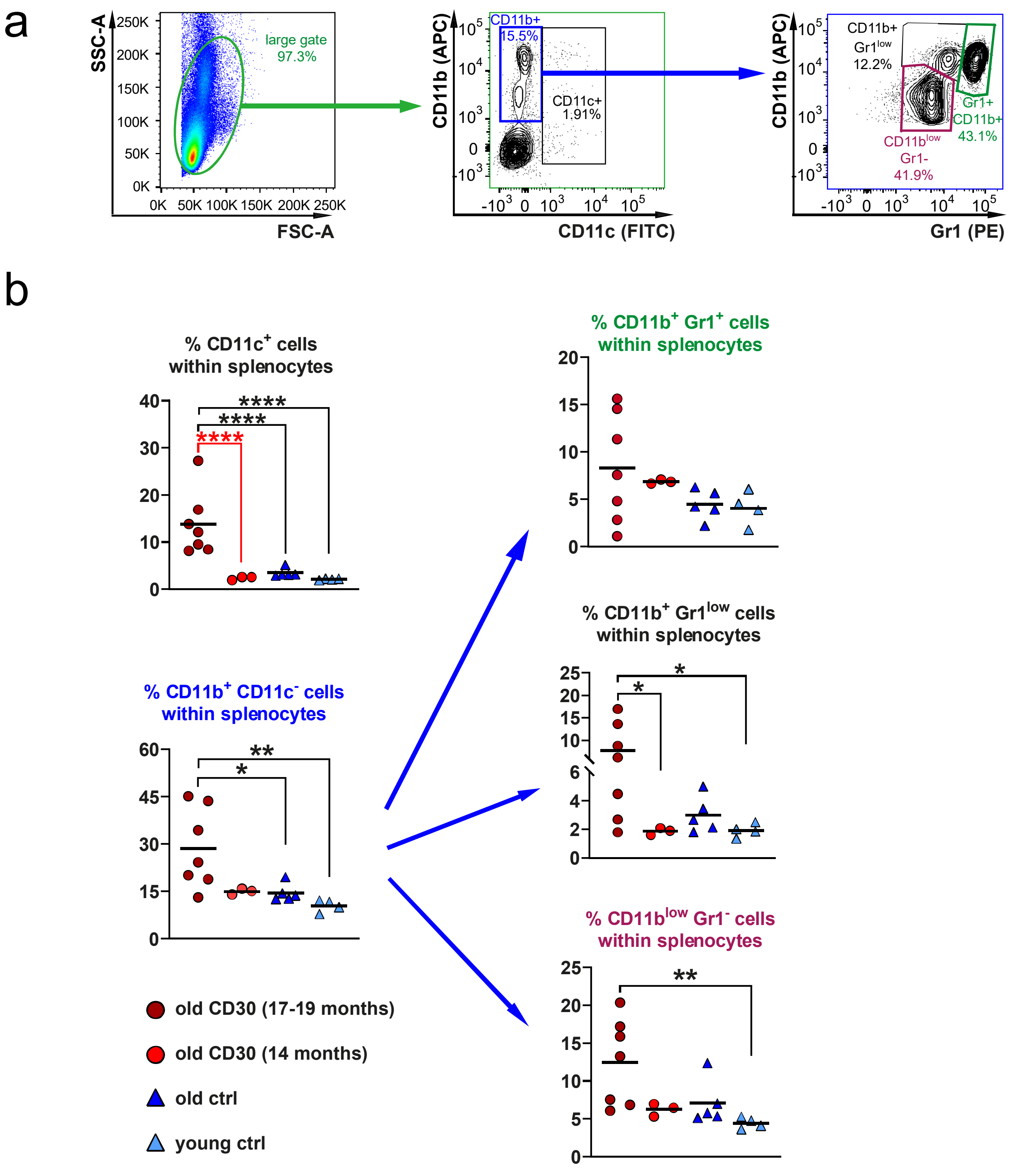

Supplementary Figure 2: Dendritic cells and myeloid subpopulations are expanded in old CD30//CD19-Cre mice with splenomegaly. a) Gating strategy for dendritic cells (DC) and myeloid subpopulations in the spleen. Cells were pre-gated on singlets and live cells as indicated in Supplementary Figure 1a. DCs and myeloid cells were gated with a large gate in a FSC/SSC plot. The gated cells were further subdivided into $CD11b^{+}CD11c^{-}$ cells and $CD11c^{+}$ cells (predominantly DCs). $CD11b^{+}CD11c^{-}$ cells were further subdivided in

$CD11b^+Gr1^+$ cells (granulocytes), $CD11b^+Gr1^-$ (macrophages and monocytes) and $CD11b^{low}Gr1^-$ cells (B1b and NK cells). **b)** Graphs depicting the percentages of the indicated cell populations within splenocytes. Old CD30//CD19-Cre mice with splenomegaly (17-19 months) are compared with old CD30//CD19-Cre mice without splenomegaly (14 months) and with old (around 16 months) and young control mice without splenomegaly (10 to 16 weeks) (N=3-7 mice per group). CD30//CD19-Cre mice are abbreviated with "CD30" in (b). An ordinary one-way ANOVA with Tukey´s multiple Comparisons test was performed. $^*P \leq 0.05$, $^{**}P \leq 0.01$, $^{****}P \leq 0.0001$.

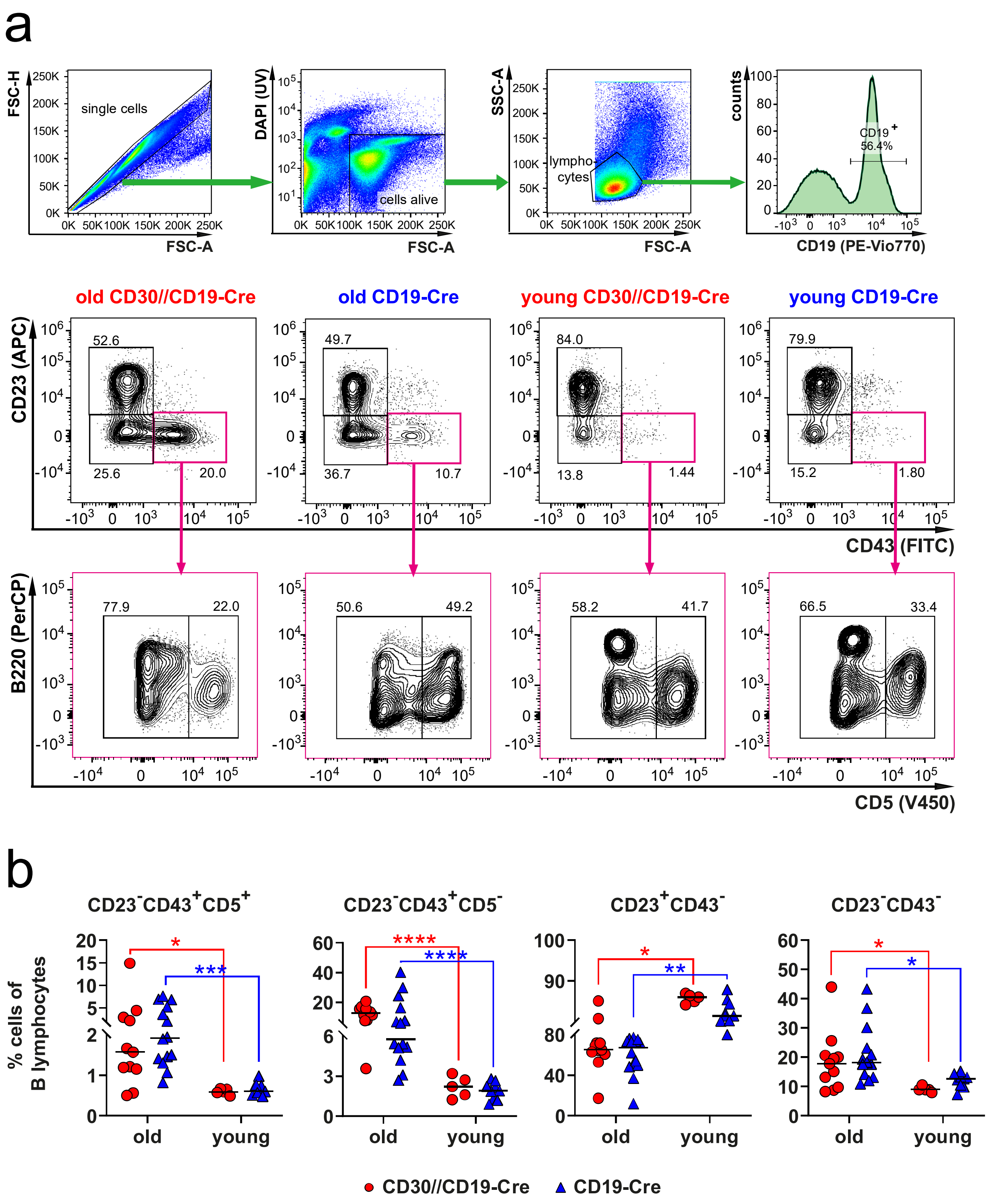

Supplementary Figure 3: The percentages of the indicated B cell populations change significantly during aging in both CD30//CD19-Cre and control mice (a) The FACS plots are sequentially pre-gated on alive single cells, lymphocytes, and CD19^{+} B cells. The

following B cell populations were gated: FoB cells + T2 cells ($CD23^+CD43^-$), MZB cells + T1 cells ($CD23^-CD43^-$), B1 cells + plasmablasts ($CD23^-CD43^+$). B1 cells were further subdivided into B1a ($CD5^+$) and B1b + activated B2 cells ($CD5^-$). (**b**)The percentages of the different B cells populations from old and young CD30//CD19-Cre (red) and control mice (blue) are summarized in the graphs (N=5-14 mice per group). $CD23^+CD43^-$ population, nonparametric Mann-Whitney test; all other populations, 2-way ANOVA with Sidak´s multiple comparisons test. *P ≤ 0.05, **P ≤ 0.01, ***P ≤ 0.001, ****P ≤ 0.0001.

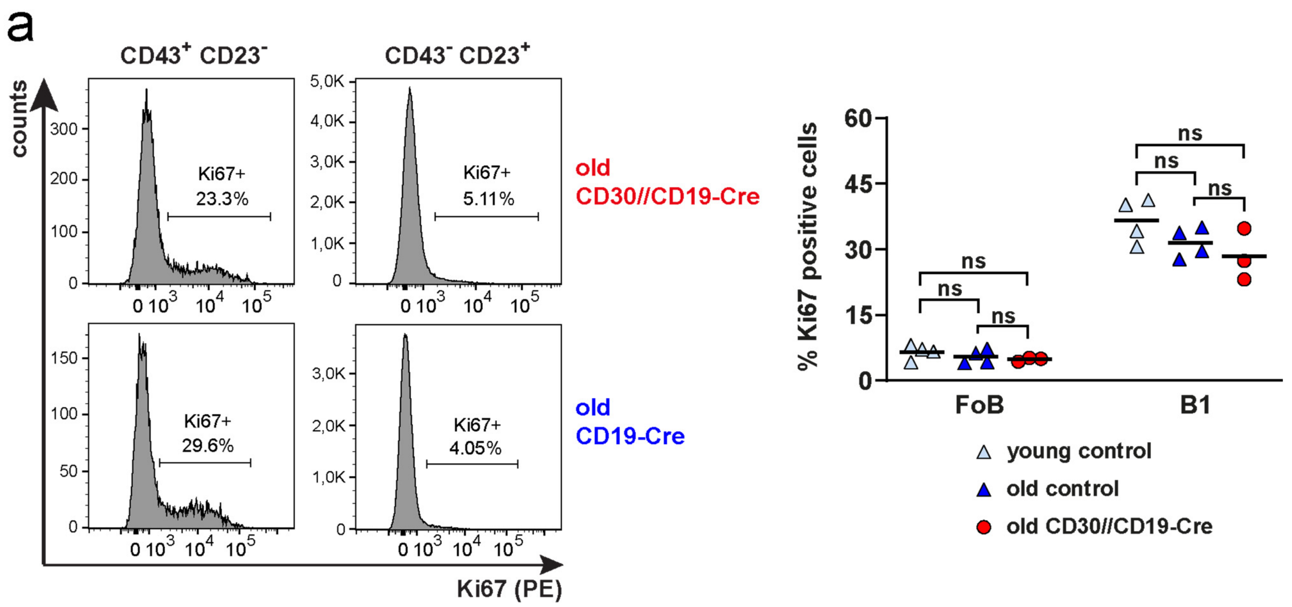

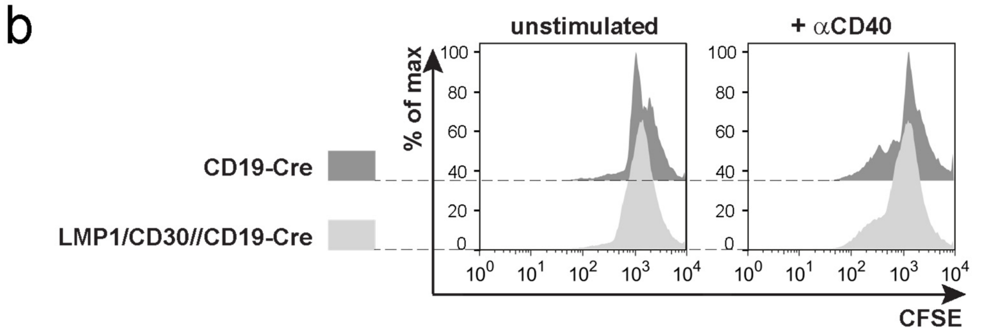

	unstimulated			+ αCD40		
	% divided	proliferation index	division index	% divided	proliferation index	division index
CD19-cre	3,31	1,83	0,06	9,63	1,52	0,15
LMP1/CD30//CD19-cre	3,93	1,28	0,05	6,54	1,83	0,12

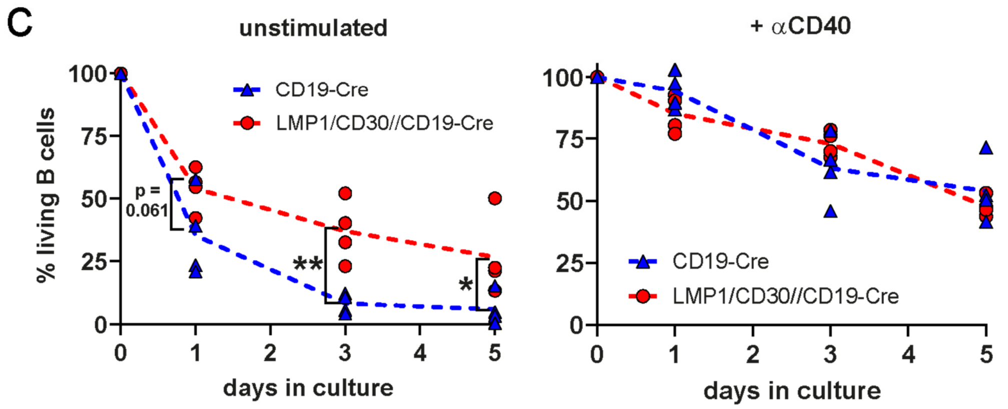

Supplementary Figure 4: CD30 signaling rather enhances survival than proliferation

(a) No genotype specific differences of the percentages of Ki67$^+$ cells between genotypes: A representative histogram demonstrating percentages of Ki67$^+$ B cells in the fraction of CD43$^+$CD23$^-$ B1 cells (left) and CD43$^-$CD23$^+$ FoB cells (right) of aged CD30//CD19-Cre

(first row) and aged control mice. Cells were pregated as shown in Supplementary Figure 3a. The graph at the right side illustrates the percentages of Ki-67$^+$ cells in different mice including also young control mice. The data were acquired in three independent experiments. (N=3-4 mice per group). (**b + c)** Proliferation and survival analyses of *ex vivo* isolated B lymphocytes from mice with constitutive CD30 signal. To determine if B cell expansion in CD30//CD19-Cre mice is dependent on increased cell division and/or improved survival, we analyzed *ex vivo* isolated splenic B lymphocytes from CD19-Cre (control) and LMP1/CD30 (LMP1/CD30//CD19-Cre) mice. We took this second mouse strain, since *ex vivo* isolated B cells from this strain are – in contrast to CD30//CD19-Cre mice - CD30L independent and deliver constitutive CD30 signaling by the LMP1/CD30 fusion protein (Sperling et al. (2019); https://doi.org/10.1182/blood.2018880138). Splenic CD19$^+$ B cells from both genotypes were MACS-purified and cultivated either unstimulated or with CD40 stimulation for 5 days. All the methods for this figure are described in detail in Hojer et al. (2014); https://doi.org/10.1158/0008-5472.CAN-13-3274). **(b)** Cells were stained with CFSE prior to stimulation. The histogram illustrates the CFSE intensity after 3 days of ex vivo culture from B cells with the indicated genotype with or without CD40 stimulation. The figure is representative for 4 independent experiments. Detailed analysis with FlowJo (see table with characteristic proliferation parameters) revealed no difference in the proliferation behavior between cells with (LMP1/CD30//CD19-Cre) and without (CD19-Cre) constitutive CD30 signaling. Data are from 4 independent experiments (4 mice per genotype). **(c)** Graphs depicting the percentages of living cells during culture compared with their numbers at the start of cultivation (set to 100%). At the indicated days cells were stained with TO-PRO-3 and analysed by flow cytometry. Without CD40 stimulation (left side) control cells die quickly. In contrast, cells from LMP1/CD30 mice show a significantly improved survival on days 3 and 5. On the right side, the graph shows the survival in the presence of CD40 stimulation which improves the survival of both LMP1/CD30 expressing cells and control cells. (N=4 mice per genotype). **a**, an ordinary one-way ANOVA with Tukey´s multiple comparisons test was performed. No significant (ns) differences were detected within the same cell populations. **c**, an ordinary two-way ANOVA with Sidak´s multiple comparison test was performed. *$p < 0.05$; **$p < 0.01$.

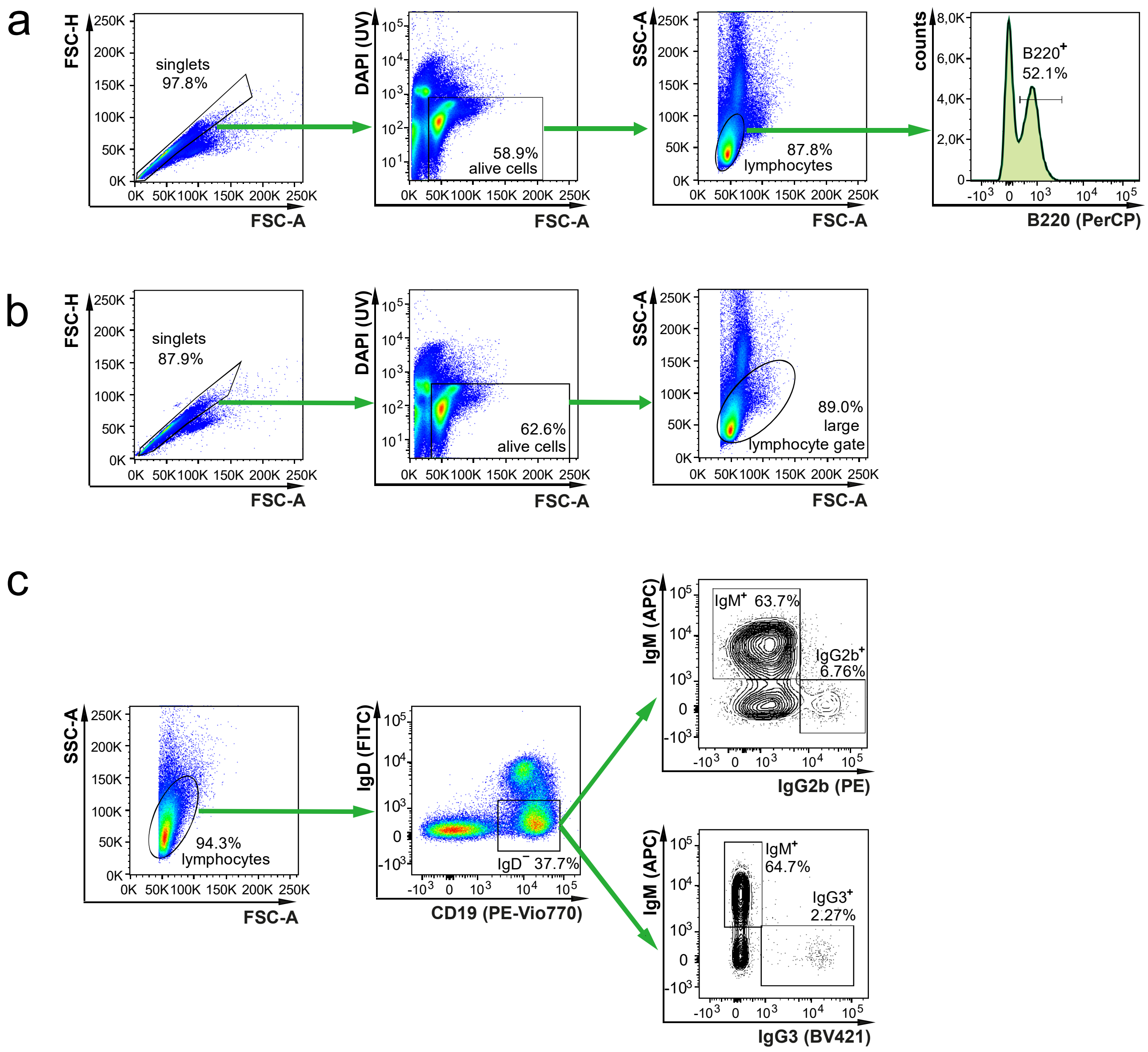

Supplementary Figure 5: gating strategies for GC B cells, PC cells and isotype switched cells (a) Exemplary pre-gating for GC B cells: Cells were subsequently gated on singlets, live cells, lymphocytes and B220^{+} cells. (**b**) Exemplary pre-gating for PC cells: Cells were subsequently gated on singlets and live cells. Subsequently an enlarged lymphocyte gate was set in a FSC/SSC plot. (**c**) Exemplary pre-gating for isotype-switched memory B cells: Cells were subsequently gated on singlets, live cells and lymphocytes as shown in (a). Afterwards cells were gated as IgD^{-}CD19^{+}. Isotype switched cells were gated as IgM^{-}IgG1^{+} (as shown in Figure 3c), IgM^{-}IgG2b^{+} or IgM^{-}IgG3^{+}.

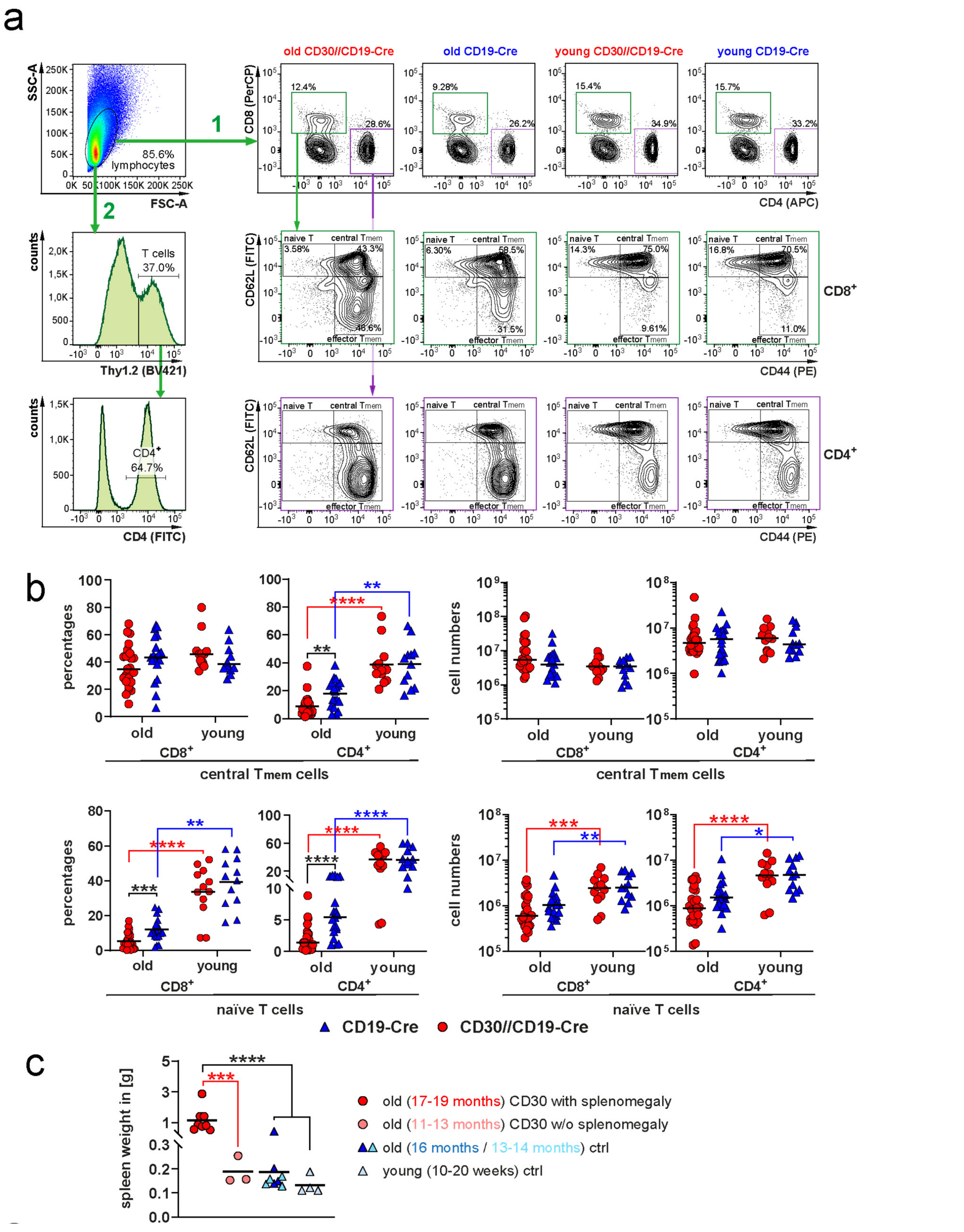

Supplementary Figure 6: T cell subpopulations in CD30//CD19-Cre and control mice:

a) Exemplary pre-gating for naïve T cells, central memory T cells and effector T cells (green arrow 1) and SA-T and Tfh cells (green arrow 2). In both cases the plots were sequentially

pre-gated on singlets, live cells and lymphocytes as shown in Supplementary Figure 5a. (1) $CD4^+$ and $CD8^+$ cells were separated in a FACS plot and then further divided based on their CD62L and CD44 expression into naïve T cells ($CD44^-CD62L^+$), central memory T cells ($CD44^+CD62L^+$) and effector T cells ($CD44^+CD62L^-$). (2) SA-T (Figure 4b) and Tfh cells (Figure 4c) were pre-gated as $Thy1.2^+$ and $CD4^+$ **(b)** Percentages and total cell numbers of naïve T cells and central memory T cells in the subsets of $CD4^+$ and $CD8^+$ T cells in young and aged CD30//CD19-Cre (red) and CD19-Cre (blue) mice are shown in graphs. (N=12-25). **(c)** Splenic weight of mice that were used to determine the percentage of CD30-L expressing SA-T and Tfh cells. CD30//CD19-Cre mice were abbreviated as CD30 in (c). (N=3-8 mice per group). **b**, 2-way ANOVA with Tukey´s multiple comparisons test. **c**, ordinary one-way ANOVA with Tukey´s multiple comparisons test. $^*P \leq 0.05$, $^{**}P \leq 0.01$, $^{***}P \leq 0.001$, $^{****}P \leq 0.0001$.

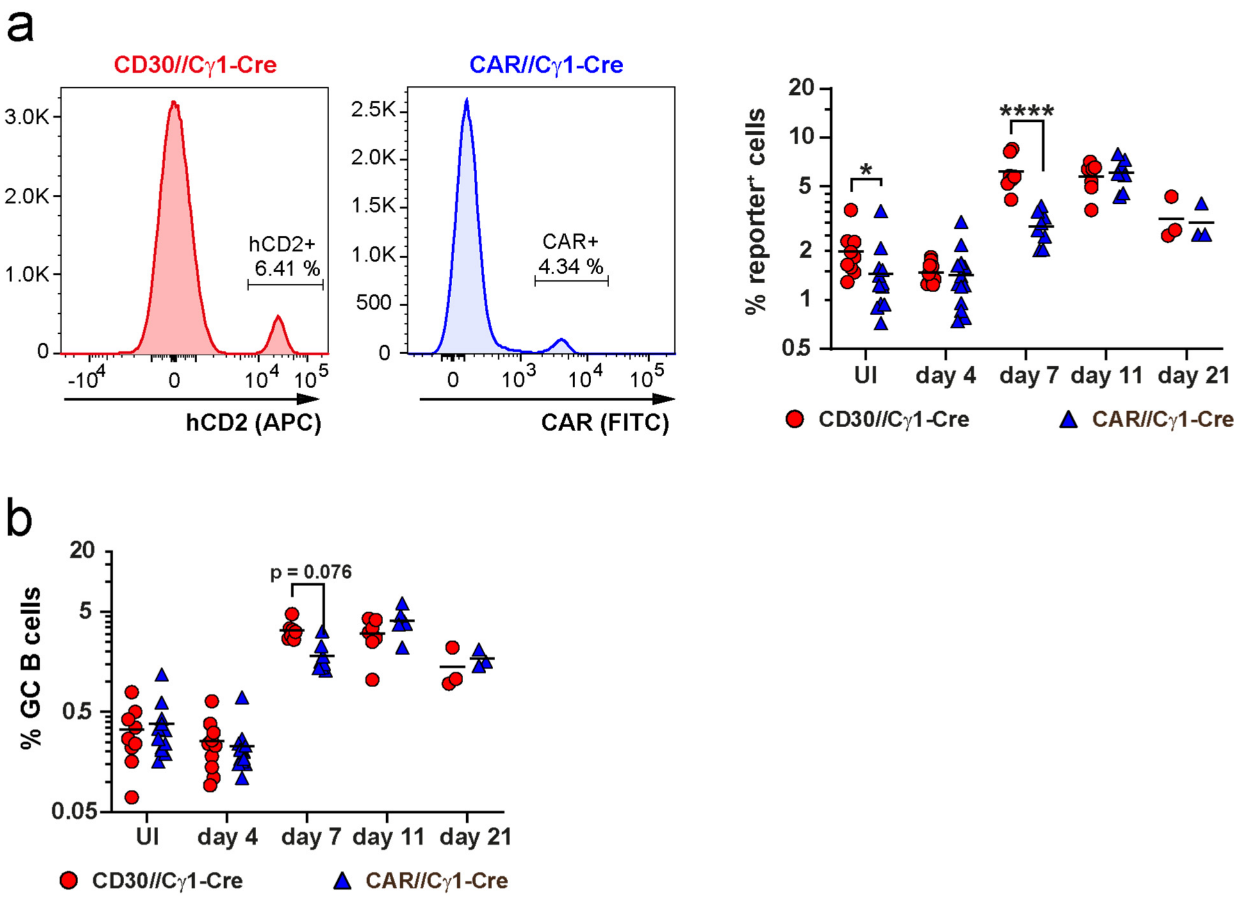

Supplementary Figure 7: The GC kinetics is accelerated in CD30//Cγ1-Cre mice in comparison to controls. (a) Exemplary gating strategy of reporter$^+$ B cells (hCD2 in CD30//Cγ1-Cre mice and CAR in CAR//Cγ1-Cre mice) from day 7 p.i.. The FACS histograms were sequentially pre-gated on singlets, living cells, lymphocytes, B220$^+$ cells (as shown in Supplementary Figure 5a). The graph summarizes the percentages of reporter$^+$ B cells in CD30//Cγ1-Cre mice (hCD2$^+$) (red dots) and controls (CAR$^+$) (blue triangles) at the indicated days after immunization in comparison to the unimmunized controls (UI) (N=3-13 per group). **(b)** The graph compiles percentages of GC B cells in indicated genotypes at specified time points after immunization (N=3-13 per group). The histogram plots are pre-gated on living B lymphocytes. **a+b**, 2-way ANOVA with Sidak´s multiple comparisons test. $^*P \leq 0.05$, $^{****}P \leq 0.0001$.

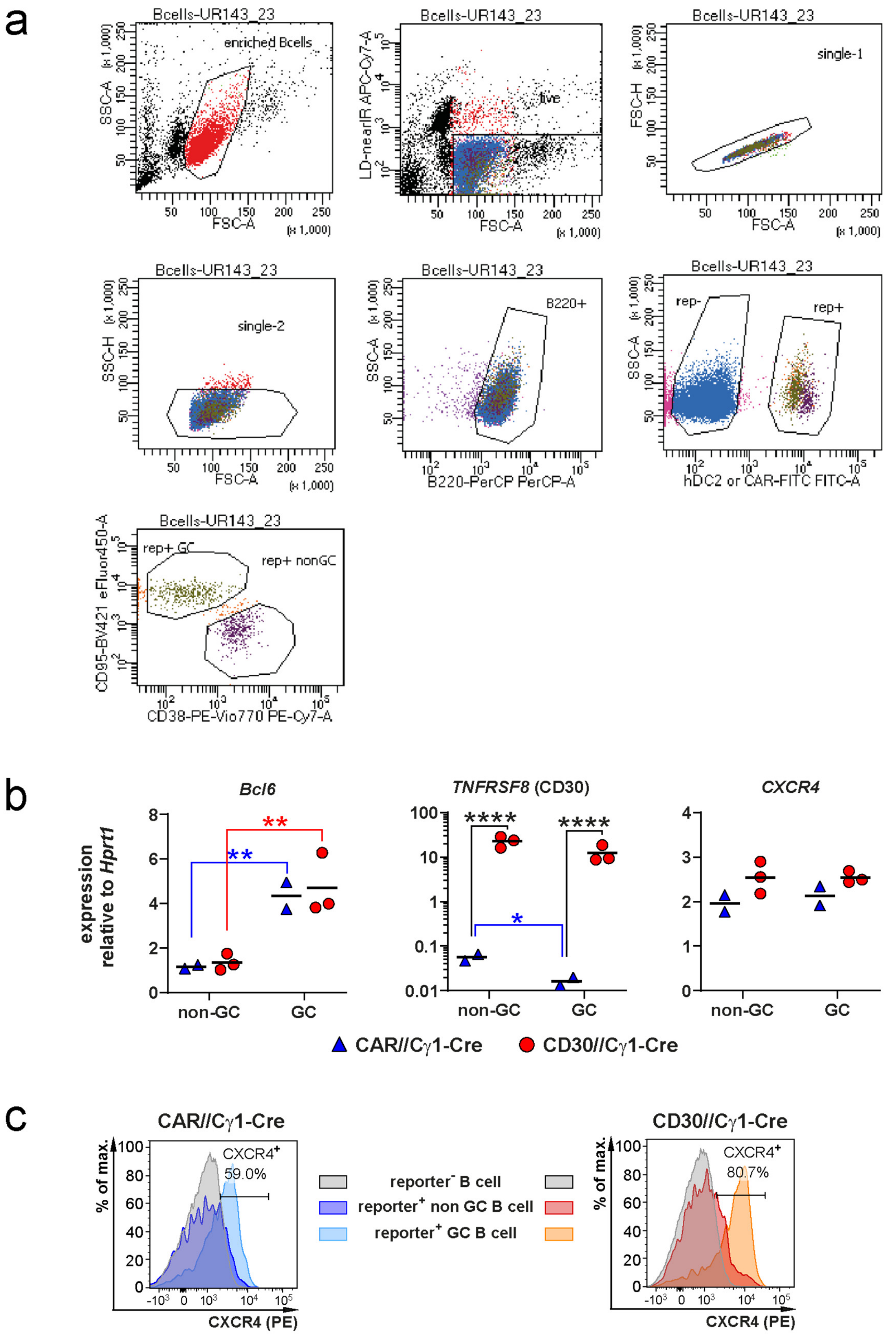

Supplementary Figure 8: *Cxcr4* mRNA levels in sorted reporter$^+$ GC and reporter$^+$ non-GC B cells: (a) Sorting strategy of reporter$^+$ GC and non-GC B cells: MACS-enriched splenic B cells were pre-gated on single, live, B220$^+$ lymphocytes. Splenocytes from CD30//Cγ1-Cre

mice and from CAR//Cγ1-CAR mice were sorted at day 11 after immunization. B cells were MACS-purified using a CD43-depletion kit prior to sorting. **(b)** mRNA levels: *Cxcr4* mRNA levels are comparable in reporter$^+$ GC and non-GC cells and between CD30//Cγ1-Cre and control mice. As control *Bcl6* mRNA expression in reporter$^+$ GC and non-GC cells was determined. *Bcl6* is higher expressed in GC than in non-GC cells. In addition, we show the higher *Tnfrsf8* (CD30) mRNA expression in reporter$^+$ GC and reporter$^-$ non-GC cells from CD30//Cγ1-Cre mice in comparison to CAR//Cγ1-CAR mice. All mRNA levels were normalized to *Hprt1* expression. (N=2-3 per genotype). 2-way ANOVAs with Tukey´s multiple correction were performed. **P ≤ 0.01, ****P ≤ 0.0001. **(c)** FACS overlay for the CXCR4 expression in reporter– cells (grey), reporter$^+$ GC and reporter$^+$ non-GC cells from CD30//Cγ1-Cre and CAR//Cγ1-Cre (control) mice 11 days post-immunization. The histogram plots are pre-gated on singlets, live cells, lymphocytes and B220$^+$ cells as shown in Supplementary Figure 5a and on reporter$^+$ GC and non-GC cells as shown in Figure 5c.

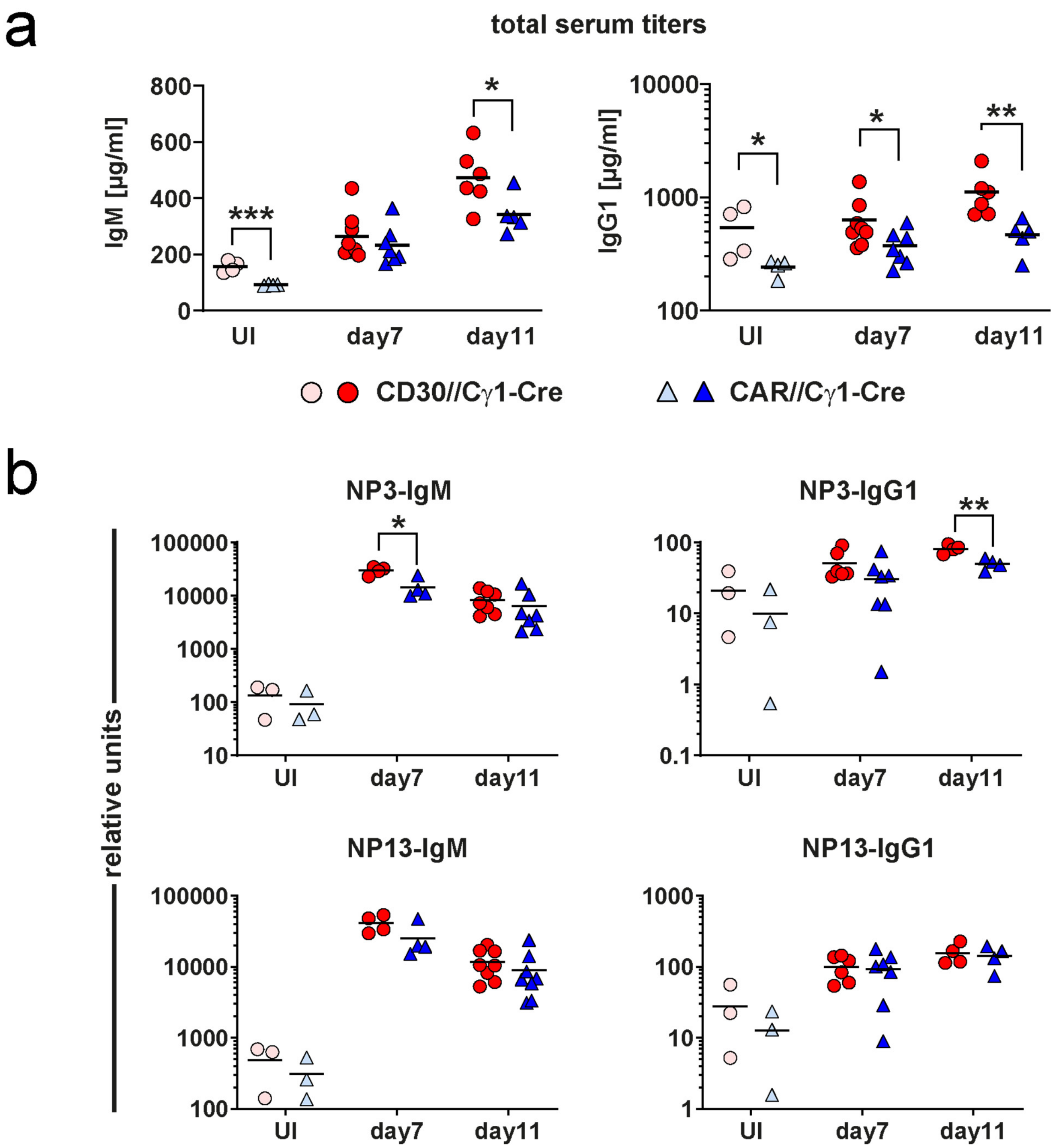

Supplementary Figure 9: Serum titers are elevated in CD30//Cγ1-Cre mice (a) total IgM and IgG1 antibodies in unimmunized (ui) and immunized (7 days and 11 days post-immunization) CD30//Cγ1-Cre and CAR//Cγ1-Cre control mice. **(b)** Relative units of NP-specific high affinity (NP3) and total (NP13) NP-specific IgM and IgG1 antibodies in the serum of CD30//Cγ1-Cre (red) and control mice (blue) 7 days or 11 days after immunization. (N=3-8 per group). Only genotypes within the same days were compared: Unpaired two-tailed t test. *P ≤ 0.05, **P ≤ 0.01, ***P ≤ 0.001.

Supplementary Table 1:

**Flow Cytometry**						
Antibody	Conjugate	Clone	Company	Catalog No.	Dilution	RRID / access link for validation data
Anti-Human CD2 (hCD2)	APC	RPA-2.10	eBioscience	17-0029-42	1:100	AB_10805740
Anti-Human CD2 (hCD2)	PE	RPA-2.10	eBioscience	12-0029-42	1:100	AB_10670621
Anti-Human CD2 (hCD2)	FITC	REA972	Miltenyi Biotec	130-116-251	1:50	AB_2727364
Anti-human CD2 (hCD2)	Pe Vio770	REA972	Miltenyi Biotec	130-116-254	1:100	AB_2727364
Anti-Human CAR	FITC	E1-1	Santa Cruz	sc-56892 FITC	1:25	10.3390/cells11050841; 10.1182/blood.2018880138;
Anti-Mouse TACI	APC	ebio8F10-3	eBioscience	17-5942	1:100	AB_842758
Anti-Mouse CD86	APC	GL1	BD Biosciences	561964	1:100	AB_2075114
Anti-Mouse CD184 (CXCR4)	PE	2B11	eBioscience	12-9991-82	1:400	AB_891391
Anti-Mouse CD38	Pe Vio770	REA616	Miltenyi Biotec	130-125-522	1:100 (1:50 for IC)	AB_2802049
Anti-Mouse CD21/CD35	BV421	7G6	BD Biosciences	562756	1:200	AB_2737772
Anti-Mouse CD138	BV421	281-2	BD Biosciences	562610	1:200	AB_11153126
Anti-Mouse CD95	BV421	Jo2	BD Biosciences	562633	1:200 (1:100 for IC)	AB_2737690
Anti-Mouse CD95	PE	Jo2	BD Biosciences	554258	1:300	AB_395330
Anti-Mouse Bcl-6	Alexa Fluor 647	K112-91	BD Biosciences	561525	1:50	AB_10898007

Anti-Mouse PRDM1/Blimp-1	PE	6D3	Santa Cruz	sc-47732 PE	1:50	https://datasheets.scbt.com/sc-47732.pdf
Anti-Mouse IRF4	eFluor 660	3E4	eBioscience	50-9858-82	1:100	AB_2574393
Anti-Mouse IRF4	PE	3E4	eBioscience	12-9858-82	1:500	AB_10852721
Anti-Mouse CD43	Biotin	S7	BD Biosciences	553269	1:350	AB_2255226
Anti-Mouse CD43	BV421	S7	BD Biosciences	562958	1:200	AB_2665409
Anti-Mouse CD19	Pe Vio770	REA749	Miltenyi Biotec	130-112-037	1:200	AB_2655830
Anti-Mouse CD19	BV510	1D3	BD Biosciences	562956	1:200	AB_2737915
Anti-Mouse CD45R/B220	PerCP	RA3-6B2	BD Biosciences	553093	1:100	AB_394622
Anti-Mouse CD45R/B220	APC	RA3-6B2	BD Biosciences	553092	1:250	AB_398531
Anti-Mouse CD45R/B220	PE	RA3-6B2	BD Biosciences	553090	1:350	AB_394619
Anti-Mouse CD45R/B220	FITC	RA3-6B2	BD Biosciences	553088	1:200	AB_394618
Streptavidin	PerCP	N/A	BD Biosciences	554064	1:100	AB_2336918
Streptavidin	APC	N/A	BD Biosciences	554067	1:400	AB_10050396
Streptavidin	BV421	N/A	BD Biosciences	563259	1:200	AB_2869475
Anti-Mouse CD23	PE	B3B4	BD Biosciences	553139	1:200	AB_394654
Anti-Mouse CD23	Alexa Fluor 647	B3B4	BD Biosciences	562826	1:100	AB_2737821
Anti-Mouse CD23	PE-Cy7	B3B4	BD Biosciences	562825	1:200	AB_2737820
Anti-Mouse IgM	APC	II/41	BD Biosciences	562032	1:100	AB_398464

Anti-Mouse IgM	PE-Cy7	R6-60.2	BD Biosciences	552867	1:300	AB_394500
Anti-Mouse IgD	Biotin	11-26c	eBioscience	13-5993-82	1:350	AB_466860
Anti-mouse IgG1	PE	A85-1	BD Biosciences	550083	1:800	AB_393553
Anti-Mouse CD184 (CXCR4)	BV510	2B11	BD Biosciences	563468	1:200	AB_2738225
Anti-Mouse CD30	FITC	mCD30.1	Invitrogen	HMCD 3001	1:80	AB_2536597
Anti-Mouse CD30	PE	mCD30.1	Invitrogen/ eBioscience	12-0301-82	1:200	AB_465629
Anti-Mouse CD5	BV450	53-7.3	BD Biosciences	561244	1:200	AB_10612004
Anti-mouse IgG1	BV450	A85-1	BD Biosciences	562107	1:200	AB_10894002
Anti-Mouse CD8a	PerCP	53-6.7	BD Biosciences	553036	1:80	AB_394573
Anti-Mouse CD4	APC	GK1.5	eBioscience	17-0041-82	1:200	AB_469320
Anti-Mouse CD4	FITC	RM4-5	BD Bioscience	553047	1:500	AB_394582
Anti-Mouse CD44	PE	IM7	BD Biosciences	553134	1:500	AB_394649
Anti-Mouse CD44	BV510	IM7	Biolegend	103043	1:100	AB_2561391
Anti-Mouse CD62L	FITC	MEL-14	BD Biosciences	553150	1:100	AB_394665
Anti-Mo/Rt Ki67	PE	SolA15	eBioscience	12-5698-82	1:500	AB_11150954
Anti-Mouse CD11c	FITC	HL3	BD Biosciences	557400	1:100	AB_396683
Anti-Mouse CD11b	APC	M1/70	Invitrogen	17-0112-81	1:500	AB_469343

Anti-Mouse CD279 (PD-1)	APC	J43	Invitrogen	17-9985-80	1:200	AB_11149358
Anti-Mouse Ly-6G and Ly-6C (Gr-1)	PE	RB6-8C5	BD Biosciences	553128	1: 200	AB_394644
Anti-Mouse IgG2b	PE	polyclonal	Invitrogen	P-21149	1:200	AB_2539818
Anti-Mouse IgG3	BV421	R40-82	BD Biosciences	565808	1:200	AB_2739364
Anti-Mouse CD153 (CD30L)	PE	RM153	Invitrogen	12-1531-82	1:100	AB_465884
Rat IgG2b k isotype control	PE	eB149/10H5	eBiosciences	12-4031-81	1:100	AB_470042
Anti-Mouse IgD	FITC	11-26c.2a	BD BioSciences	553439	1:400	AB_394859
**Histology (IF/IHC)**						
Rabbit anti-Laminin	unconjugated	L9393	Sigma-Aldrich	L9393	1:100	AB_477163
Rat anti-Mouse CD90.2 (Thy1.2)	Biotin	30-H12	BD Biosciences	553011	1:100	AB_394549
Rat anti-Mouse CD45R/B220	APC	RA3-6B2	BD Biosciences	553092	1:500	AB_398531
Rat anti-Mouse T- and B-Cell Activation Antigen (GL7)	FITC	GL7	BD Biosciences	553666	1:100	AB_394981
Rat anti-Metallophilic Macrophages (MOMA-1)	Biotin	MOMA-1	Abcam	ab51814	1:100	https://www.abcam.com/products/primary-antibodies/biotin-metallophilic-macrophages-antibody-moma-1-ab51814.html
Rat anti-Mouse Irf4	unconjugated	3E4	eBioscience	14-9858-80	1:100	https://www.thermofisher.com/antibody/product/IRF4-Antibody-clone-

						3E4-Monoclonal/14-9858-82
Goat anti-Rat IgG	Alexa Fluor 488	N/A	Jackson ImmunoResearch	112-545-003	1:500	AB_2338351
Goat anti-rabbit IgG	Cyanine Cy3	N/A	Jackson ImmunoResearch	111-165-003	1:500	AB_2338000
Streptavidin	Alexa Fluor 594	N/A	Invitrogen	S11227	1:500	https://www.thermofisher.com/order/catalog/product/de/en/S11227
Peanut Agglutinin (PNA)	Biotin	PNA	Vector	B-1075-5	1:2000	https://vectorlabs.com/products/biotinylated-peanut-agglutinin-pna
**Western Blot**						
CD30 Antibody (C-3)	unconjugated	C-3	Santa Cruz	sc-46683	1:100	
Anti-mouse IgG, HRP-linked Antibody	HRP		Cell Signaling	#7076	1:2000	
**Enzyme-linked Immunosorbent Assay (ELISA)**						
rat-anti-mouse IgM	purified	II/41	BD Biosciences	553435	1:100	AB_394855
rat-anti-mouse IgG1	purified	A85-3	BD Biosciences	553445	1:100	AB_394863
mouse IgM	purified	G155-228	BD Biosciences	553472	1:400	AB_395957
mouse IgG1	purified	MOPC-31C	BD Biosciences	557273	1:400	AB_396613
rat-anti-mouse IgM	HRP	YF97	Southern Biotech	1140-05	1:5000	AB_2794629
rat-anti-mouse IgG1	Biotin	A85-1	BD Biosciences	553441	1:500	AB_394861
Avidin D	HRP	N/A	Vector	A-2014-5	1:2000	https://vectorlabs.com/products/avidin-d-horseradish-peroxidase-concentrate-elisa
Streptavidin, Alkaline Phosphatase	AP	N/A	Vector	SA-5100	1:1000	https://vectorlabs.com/products/streptavidin-alkaline-phosphatase-conjugated

Supplementary Table 2: Primer for qRT PCR

Gene Name	GenBank	Primer forward	Primer reverse	Probe (#)
Tnfrsf8 (*Cd30*)	NM_009401	AAGTACCGCCTCCACCCTAA	TGGTGTGTTCCGTGGATACC	25
Cxcr4	NM_009911	GGACCGGTACCTCGCTATTG	TCCACAGGCTATCGGGGTAA	63
Bcl6	NM_009744	CCTGAAGACCCACACTCGAA	ACACGCGGTATTGCACCTTG	10
Hprt1	NM_013556	TGATAGATCCATTCCTATGACTGTAGA	AAGACATTCTTTCCAGTTAAAGTTGAG	95
